# PICK1 regulates AMPA receptor endocytosis via direct interactions with AP2 α-appendage and dynamin

**DOI:** 10.1083/jcb.201701034

**Published:** 2017-10-02

**Authors:** Maria Fiuza, Christine M. Rostosky, Gabrielle T. Parkinson, Alexei M. Bygrave, Nagaraj Halemani, Marcio Baptista, Ira Milosevic, Jonathan G. Hanley

**Affiliations:** 1 Centre for Synaptic Plasticity and School of Biochemistry, University of Bristol, Bristol, England, UK; 2 European Neuroscience Institute, University Medical Center Göttingen, Göttingen, Germany

## Abstract

Clathrin-mediated endocytosis (CME) is used to internalize a diverse range of cargo proteins from the cell surface, often in response to specific signals. In neurons, the rapid endocytosis of GluA2-containing AMPA receptors (AMPARs) in response to NMDA receptor (NMDAR) stimulation causes a reduction in synaptic strength and is the central mechanism for long-term depression, which underlies certain forms of learning. The mechanisms that link NMDAR activation to CME of AMPARs remain elusive. PICK1 is a BAR domain protein required for NMDAR-dependent reductions in surface GluA2; however, the molecular mechanisms involved are unclear. In this study, we show that PICK1 makes direct, NMDAR-dependent interactions with the core endocytic proteins AP2 and dynamin. PICK1–AP2 interactions are required for clustering AMPARs at endocytic zones in dendrites in response to NMDAR stimulation and for consequent AMPAR internalization. We further show that PICK1 stimulates dynamin polymerization. We propose that PICK1 is a cargo-specific endocytic accessory protein required for efficient, activity-dependent AMPAR endocytosis.

## Abstract (precis)

Fiuza et al. report that PICK1 localizes to clathrin-coated pits and makes direct, functional interactions with the endocytic adapter complex AP2 and dynamin. The PICK1–AP2 interaction is required for clustering AMPA receptors at endocytic sites and for consequent AMPA receptor endocytosis, defining PICK1 as a cargo-specific endocytic accessory protein.

## Introduction

Clathrin-mediated endocytosis (CME) is the major mechanism for the internalization of integral membrane proteins from the cell surface before processing in the endosomal system. It is a highly orchestrated process involving numerous proteins that recruit and concentrate cargo at specific membrane domains, manipulate plasma membrane geometry to form the invaginated pit, and finally drive scission of the fully formed vesicle from the plasma membrane (McMahon and Boucrot, 2011). A central player in this process is the adapter protein complex AP2, which clusters at PI(4,5)P_2_-rich domains in the plasma membrane and binds cargo proteins, numerous endocytic accessory proteins, and clathrin (Robinson, 2004; Traub, 2009; Kelly and Owen, 2011). Several such accessory proteins, including amphiphysin, endophilin, and sorting nexin 9 (SNX9), contain a BAR domain, which senses or contributes to membrane curvature at the neck of the clathrin-coated pit (CCP), and a major role of these proteins is to recruit dynamin to this structure via SH3 domain interactions (Taylor et al., 2011; Daumke et al., 2014; Suetsugu et al., 2014). Dynamin is a large GTPase that polymerizes around the neck of the CCP and mediates scission of the endocytic vesicle via GTP hydrolysis (Ferguson and De Camilli, 2012). A wide diversity of plasma membrane proteins need to be internalized in a highly regulated manner in response to specific signals; hence, there is a requirement for mechanisms that transduce relevant upstream signaling into the rapid and efficient internalization of specifically selected cargo (Traub, 2009).

The precise regulation of AMPA receptor (AMPAR) trafficking in neurons is crucial to excitatory neurotransmission, synaptic plasticity, and the consequent formation and modification of neural circuits during brain development and learning (Kessels and Malinow, 2009; van der Sluijs and Hoogenraad, 2011; Huganir and Nicoll, 2013). Furthermore, AMPAR trafficking is affected in a range of neurological disorders, including Alzheimer’s, Huntington’s, and brain ischemia, among others (Henley and Wilkinson, 2016). CME is an essential trafficking event for the activity-dependent removal of AMPARs from the neuronal plasma membrane, resulting in a reduction in synaptic strength known as long-term depression (LTD; Man et al., 2000; Anggono and Huganir, 2012). The regulated AMPAR endocytosis that underlies LTD is caused by specific modes of synaptic activity, most notably NMDA receptor (NMDAR) stimulation (Beattie et al., 2000; Huganir and Nicoll, 2013). Although it is known that NMDAR-dependent AMPAR endocytosis requires dynamin and AP2 (Man et al., 2000; Lee et al., 2002), the molecular mechanisms that mediate the transduction of NMDAR stimulation into modulation of these core endocytic proteins to efficiently drive AMPAR endocytosis remain elusive. In particular, the identity and precise function of endocytic accessory proteins that perform this role are unknown.

PICK1 is a PDZ and BAR domain–containing protein that interacts with the AMPAR subunit GluA2. The GluA2–PICK1 interaction is enhanced by direct binding of Ca^2+^ ions to PICK1 in a mechanism that is required for LTD (Hanley and Henley, 2005; Citri et al., 2010). Although PICK1 function is known to result in the intracellular accumulation of plasma membrane–derived, GluA2-containing AMPARs, previous evidence suggests a role in restricting postendocytic recycling back to the plasma membrane and not in CME per se (Lin and Huganir, 2007; Citri et al., 2010; Widagdo et al., 2016). However, we noticed that PICK1 contains sequence motifs conforming to AP2 appendage domain interaction sites, similar to those found in amphiphysin and SNX9 (Praefcke et al., 2004; Olesen et al., 2008), leading to our hypothesis that PICK1 interacts with the core endocytic machinery and therefore plays a role in CME of AMPARs.

In this study, we define PICK1 as an endocytic accessory protein that associates with CCPs, is required for NMDAR-dependent targeting of GluA2-containing AMPARs to endocytic zones (EZs) in neuronal dendrites via a direct interaction with AP2, and promotes dynamin polymerization by directly binding to dynamin’s GTPase domain. These novel interactions are increased as a result of NMDAR stimulation and are essential for NMDAR-stimulated AMPAR internalization.

## Results

### PICK1 localizes to EZs in neuronal dendrites

**Figure 1.**
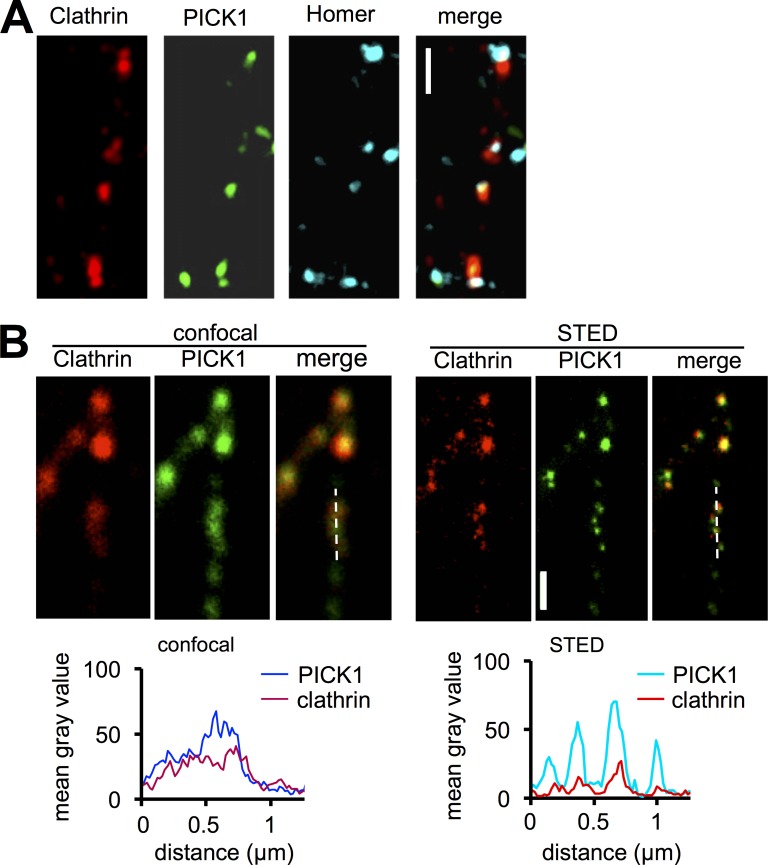
**PICK1 colocalizes with clathrin in neuronal dendrites.** (A) PICK1 colocalizes with clathrin clusters close to synapses. Confocal images of hippocampal neurons transfected with PICK1 shRNA + sh-resistant GFP-PICK1 (green) and dsRed-clathrin (red) and immunostained for the PSD marker Homer (blue). Bar, 2 µm. (B) PICK1 colocalizes with clathrin clusters under super-resolution imaging conditions. Confocal and STED images of hippocampal neurons transfected with PICK1 shRNA + sh-resistant GFP-PICK1 and dsRed-clathrin and immunostained for GFP and dsRed. Bar, 0.5 µm. Graphs show line-scan analyses of PICK1 and clathrin fluorescence intensities of dashed line regions in confocal and STED mode, demonstrating the colocalization of PICK1 and clathrin clusters. Images shown are representative of three independent experiments.

To investigate a role for PICK1 in CME in neurons, we analyzed its localization at EZs adjacent to synapses in dendrites of cultured neurons. To define EZs, we expressed dsRed-clathrin in cultured neurons (Blanpied et al., 2002) and coexpressed molecular replacement constructs comprising PICK1 shRNA and sh-resistant GFP-PICK1 to replace endogenous PICK1 with GFP-PICK1 (Citri et al., 2010; Antoniou et al., 2014). GFP-PICK1 and dsRed-clathrin showed a colocalizing clustered distribution in dendrites (Fig. 1 A), and PICK1–clathrin clusters were closely associated with the postsynaptic density (PSD) marker Homer. However, a subset of PSDs was not associated with PICK1–clathrin clusters, consistent with previous studies of the distribution of EZs (Blanpied et al., 2002; also see Fig. S2 B). To confirm the colocalization of PICK1 with clathrin, we improved spatial resolution using stimulated emission depletion (STED) super-resolution imaging. Under these imaging conditions, PICK1 showed a marked colocalization with clathrin clusters (Fig. 1 B).

### PICK1 binds AP2 via FxDxF and DxF motifs

**Figure 2.**
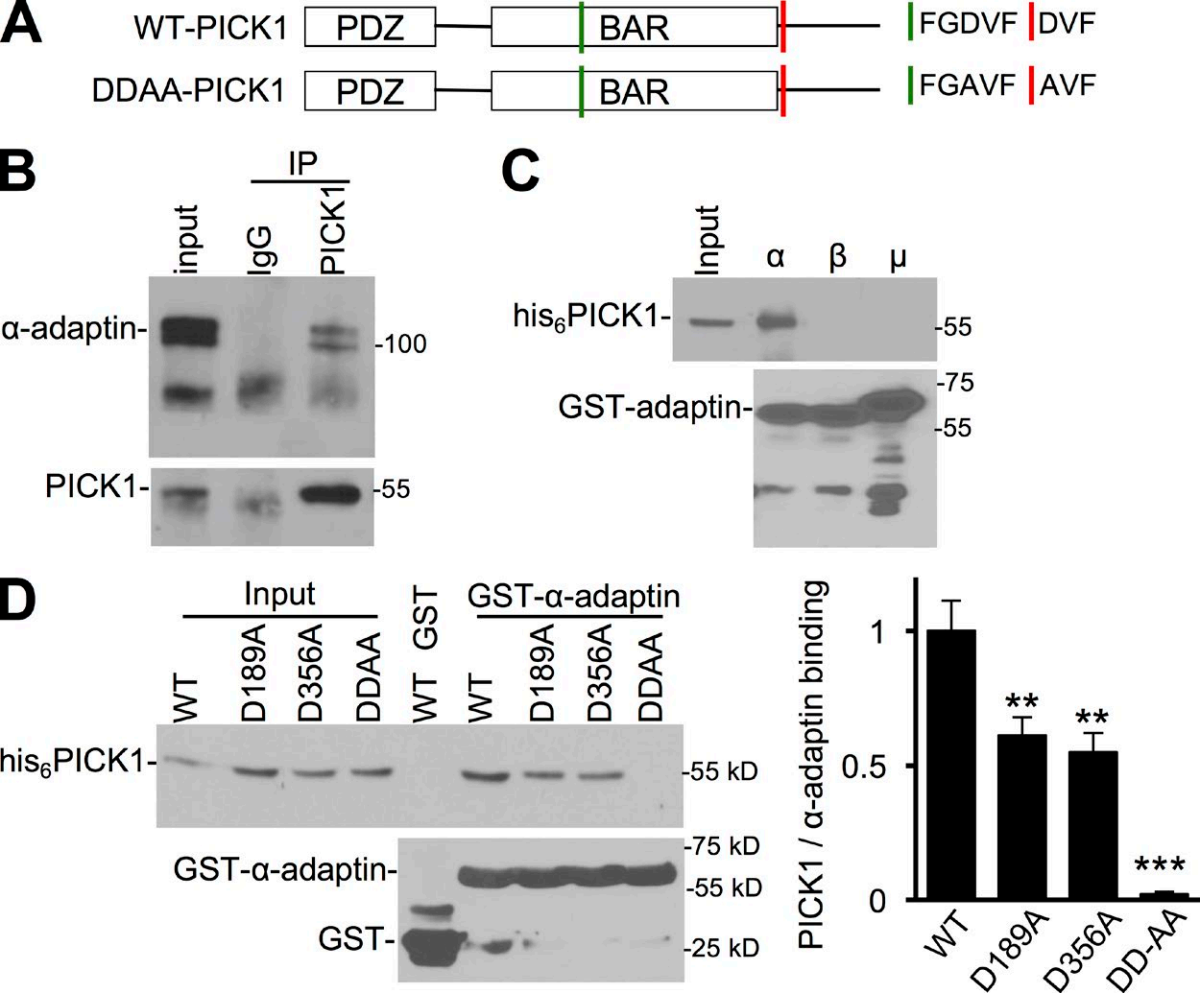
**PICK1 binds AP2 via FxDxF and DxF motifs.** (A) Schematic of PICK1 indicating the FxDxF and DxF AP2-binding motifs and the DD189,356AA mutations. (B) Endogenous PICK1 binds endogenous AP2 in neurons. Extracts of cortical neurons were immunoprecipitated with PICK1 antibody or control IgG. Proteins were detected by Western blotting. Input is 5% of offered protein. Blots shown are representative of more than five independent experiments. (C) PICK1 directly interacts with the appendage domain of α-adaptin. GST, GST-α-appendage, GST-β-appendage, or GST-μ cargo-recognition domain were immobilized on glutathione agarose and incubated with purified his_6_-PICK1. Proteins were detected by Western blotting. Blots shown are representative of five independent experiments. (D) DDAA mutant does not bind AP2. GST or GST-α-appendage were immobilized on glutathione agarose and incubated with purified his_6_-WT-PICK1 or mutants. Graph shows quantification of PICK1 binding to α-adaptin (*n* = 6 independent experiments; **, P < 0.01; ***, P < 0.001; one-way ANOVA followed by Tukey’s test; values are means ± SEMs).

Numerous endocytic accessory proteins are recruited to CCPs by interacting with AP2 α-adaptin appendage domains via FxDxF or DxF motifs. For example, amphiphysin binds AP2 via one FxDxF and one DxF motif (Praefcke et al., 2004; Olesen et al., 2008). Because PICK1 contains ^187^FGDVF^191^ and ^356^DVF^358^, which conform to these consensus sequences (Fig. 2 A), we sought to determine whether PICK1 interacts with AP2. In coimmunoprecipitations (co-IPs) from neuronal lysates, we found that α-adaptin associated robustly with PICK1 (Fig. 2 B). AP2 is a stable complex of α, β2, μ2, and σ2 adaptins (Robinson, 2004); hence, the presence of α-adaptin in the PICK1 immunopellet indicates an association with the AP2 complex. The double band observed in α-adaptin Western blots corresponds to the two α-adaptin genes expressed in neurons, αA-adaptin, and αC-adaptin (Ball et al., 1995). To define which AP2 subunit interacts with PICK1, we performed GST pulldowns with purified proteins. His_6_-PICK1 showed a robust interaction with the GST-α-appendage domain, but not the β2-appendage nor the μ2 cargo-recognition domain (Fig. 2 C). Importantly, this experiment also demonstrated that PICK1 binds directly to the AP2 complex. To investigate whether PICK1 binds α-appendage domains via ^187^FGDVF^191^ and ^356^DVF^358^, we generated two separate mutants—D189A and D356A—as well as a double mutant, DD189,356AA (hereafter referred to as DDAA). Both D189A and D356A showed markedly reduced binding to α-adaptin, and α-adaptin binding to DDAA-PICK1 was essentially abolished (Fig. 2 D). Because D189 is located in the BAR domain, which is the dimerization domain and also binds lipid membranes and F-actin (Jin et al., 2006; Rocca et al., 2008), and D356 is in the C-terminal domain, which binds the Arp2/3 complex (Rocca et al., 2008), we analyzed whether the DDAA mutations affected these binding properties of PICK1. DDAA-PICK1 showed similar dimerization, lipid, F-actin, and Arp2/3 binding properties as WT-PICK1 (Fig. S1). In neurons coexpressing GFP-DDAA-PICK1 and mCherry-WT-PICK1, both proteins showed a clustered distribution in dendrites. Although a proportion of clusters contained predominantly WT-PICK1 and others contained predominantly DDAA-PICK1, the majority of clusters contained both proteins (Fig. S2 A). PICK1 has been shown previously to colocalize with synapses as well as endosomal compartments in neurons (Sossa et al., 2006; Rocca et al., 2008). The DDAA mutation had no significant effect on PICK1 colocalization with the synaptic marker Homer in neuronal dendrites (Fig. S2 B), the early endosomal marker EEA1, or the recycling endosomal marker Rab11 (Fig. S2, C and D).

These experiments identify PICK1 as a novel AP2-interacting protein via direct binding to the α-adaptin appendage domain and define the DDAA double mutant as an appropriate tool for investigating the function of this interaction.

### PICK1 is recruited to CCPs in heterologous cells

**Figure 3.**
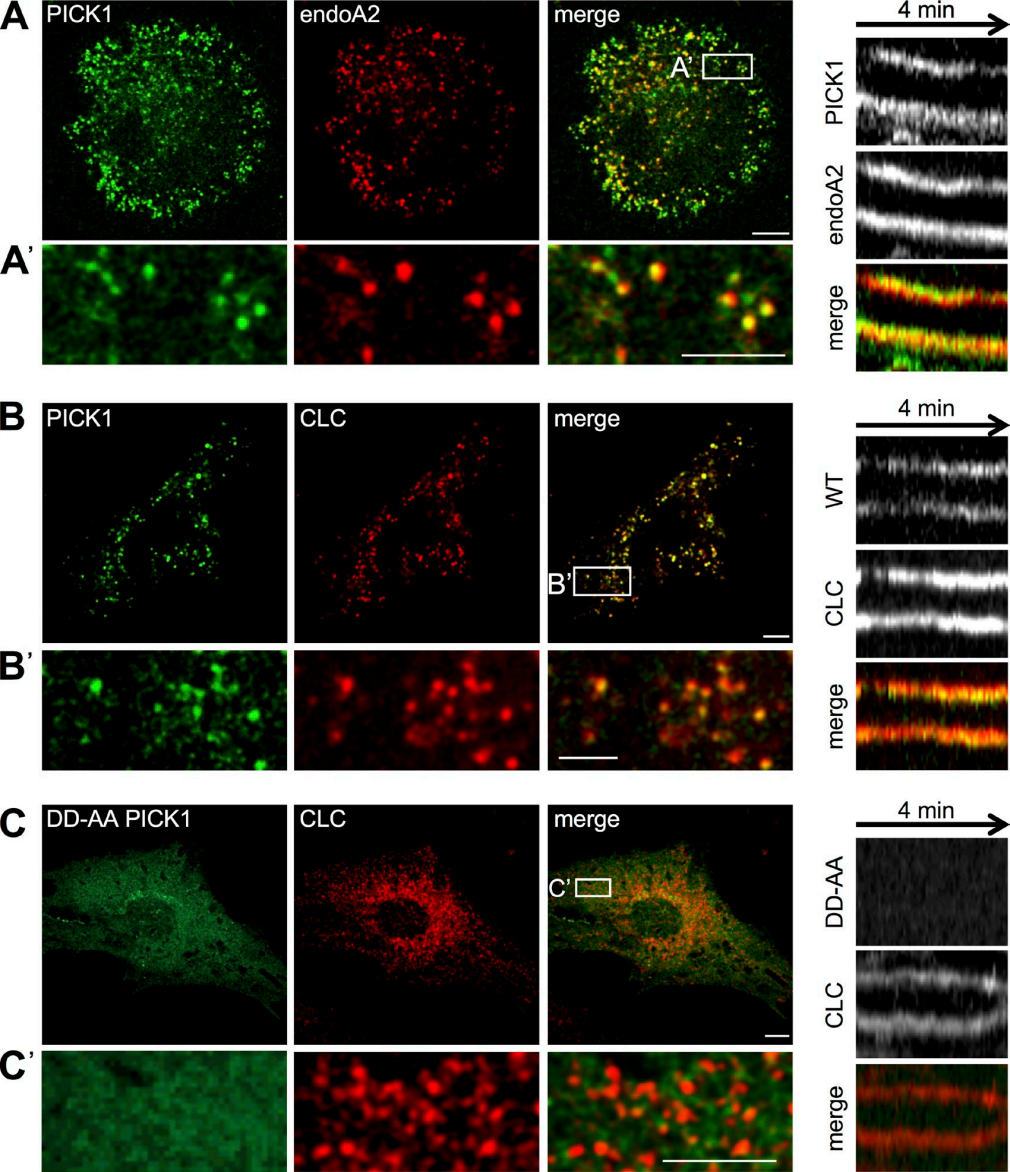
**PICK1 is recruited to CCPs in an AP2-dependent and a dynamin-independent manner in dynamin TKO fibroblasts.** (A) PICK1 is localized on arrested CCP necks labeled with endophilin-A2. Dynamin TKO fibroblasts expressing GFP-WT-PICK1 and endophilin-A2–mCherry were imaged live by spinning-disk confocal microscopy. A’ shows magnification from boxed region. (A, right) Kymograph shows that the PICK1–endophilin-A2 colocalization is stable for several minutes. (Note that these cells accumulate arrested CCPs.) (B) PICK1 colocalizes with the clathrin coat. Dynamin TKO fibroblasts expressing GFP-WT-PICK1 and clathrin LC–mRFP were imaged live by spinning-disk confocal microscopy. B’ shows magnification from boxed region. (B, right) Kymograph shows that the PICK1–clathrin LC (CLC) colocalization is stable for several minutes. (C) Localization of PICK1 to CCPs requires interaction with AP2. Dynamin TKO fibroblasts expressing GFP-DDAA-PICK1 and clathrin LC–mRFP were imaged live by confocal microscopy. C’ shows magnification from boxed region. (C, right) Kymograph shows that GFP-DDAA-PICK1 does not colocalize with clathrin LC over time. Bars: (whole cells) 10 µm; (magnified images) 5 µm.

To investigate whether PICK1 associates with CCPs, we expressed GFP-PICK1 in dynamin triple knockout (TKO) mouse embryonic fibroblasts. In the absence of dynamin, fission of clathrin-coated vesicles is blocked, causing elongated and persistent CCP necks, providing a system for studying recruitment of proteins to these structures (Ferguson et al., 2009; Milosevic et al., 2011). Endophilin-A is known to associate with CCP necks, so we cotransfected dynamin TKO cells with GFP-WT-PICK1 and endophilin-A2-mRFP. GFP-WT-PICK1 showed a marked colocalization with endophilin-A2-mRFP clusters (Fig. 3 A), strongly suggesting that PICK1 is recruited to CCP necks. The colocalization between PICK1 and endophilin-A2 usually lasted for the whole duration of recording (Video 1). Next, we coexpressed GFP-WT-PICK1 with clathrin light chain (LC)–mRFP. Interestingly, we observed a near-complete colocalization of PICK1 and clathrin, showing that PICK1 can be recruited to the clathrin coat as well as the CCP neck (Fig. 3 B; Video 2). To investigate whether the localization of PICK1 at CCPs depends on its binding to AP2, we cotransfected dynamin TKO fibroblasts with GFP-DDAA-PICK1 and clathrin LC–mRFP. The colocalization of PICK1 with CCPs was abolished by this mutation (Fig. 3 C; Video 3). Instead, most GFP-DDAA-PICK1 was diffusely distributed in the cytoplasm, with some clustering in a juxtanuclear compartment, which partially colocalized with early and recycling endosome markers (Fig. S3, A and B). Together, these data strongly suggest that PICK1 is recruited to CCPs in an AP2-dependent and dynamin-independent manner and also binds to the CCP neck.

### PICK1 binds dynamin via BAR and GTPase domains and promotes dynamin polymerization in vitro

**Figure 4.**
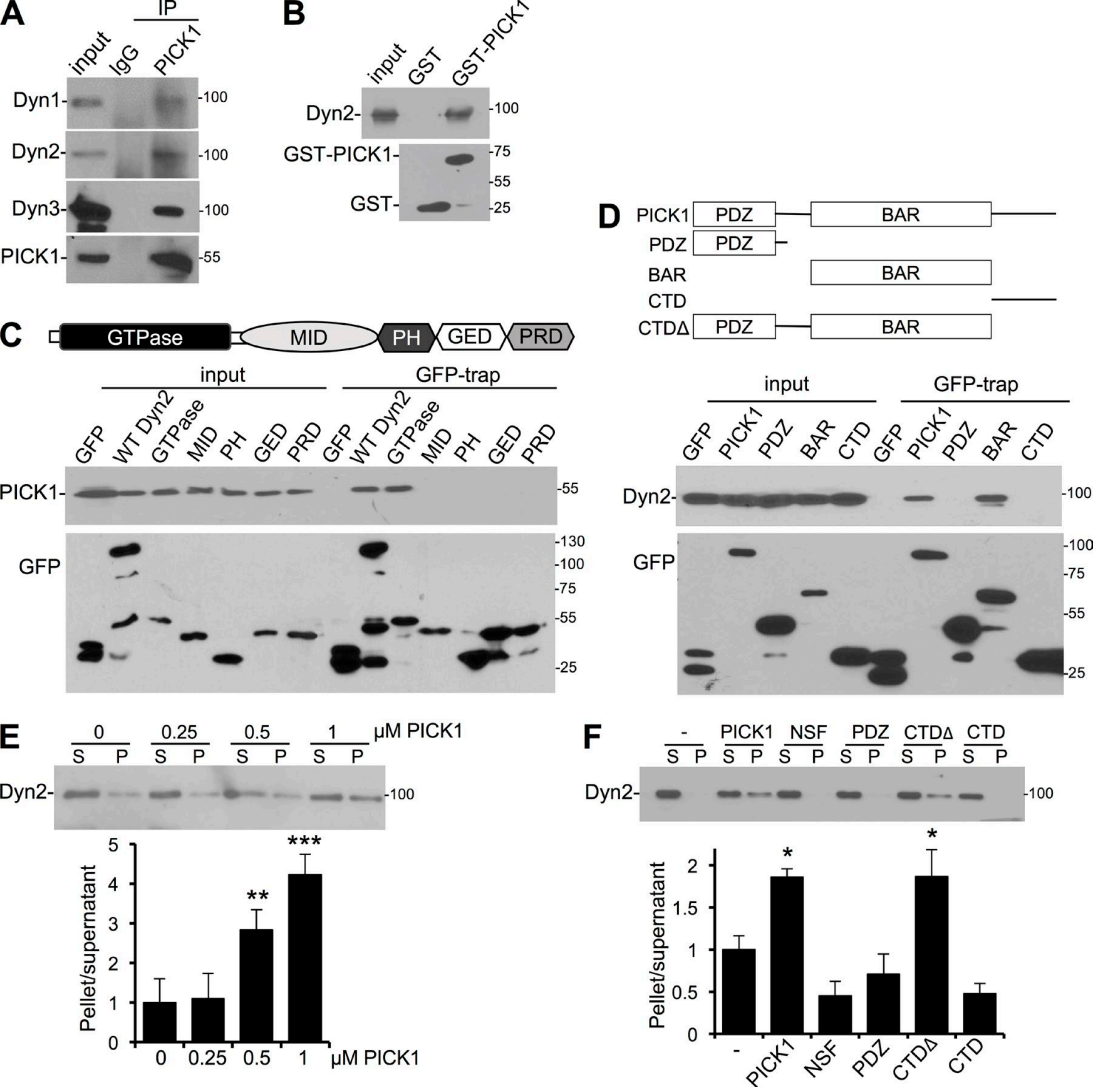
**PICK1 binds dynamin via BAR and GTPase domains and regulates dynamin polymerization.** (A) Endogenous PICK1 interacts with endogenous dynamin 1, 2, and 3 in neurons. Extracts of cortical neurons were immunoprecipitated with PICK1 antibody or control IgG. Proteins were detected by Western blotting. Input is 5% of offered protein. Blots shown are representative of more than five independent experiments. (B) PICK1 interacts directly with dynamin. GST or GST-PICK1 was immobilized on glutathione agarose and incubated with purified HA-dynamin. Proteins were detected by Western blotting. Blots shown are representative of four independent experiments. (C) PICK1 interacts with the GTPase domain of dynamin. HEK293 cells were cotransfected with plasmids expressing Flag-PICK1 and GFP, GFP–dynamin 2, or dynamin domains as indicated. Cells were lysed and incubated with GFP-trap agarose. Blots shown are representative of five independent experiments. (D) Dynamin interacts with the PICK1 BAR domain. HEK cells were cotransfected with plasmids expressing HA-dynamin and GFP, GFP-PICK1, or truncations, as indicated in the top panel. Cells were lysed and incubated with GFP-trap agarose. Blots shown are representative of five independent experiments. (E) PICK1 promotes dynamin polymerization. His_6_-PICK1 at the concentrations indicated was incubated with HA-dynamin. Polymerized dynamin was pelleted by centrifugation, and protein in the supernatant and pellet was analyzed by Western blotting. Graph shows the ratio of dynamin in pellet/supernatant (*n* = 4 independent experiments; **, P < 0.01; ***, P < 0.001; one-way ANOVA followed by Tukey’s test; values are means ± SEMs). (F) HA-dynamin was incubated with his_6_-PICK1, his_6_-NSF, or his_6_-PICK1 truncations as indicated and processed as in E. Graph shows the ratio of dynamin in pellet/supernatant (*n* = 7 independent experiments; *, P < 0.05; one-way ANOVA followed by Tukey’s test; values are means ± SEMs). CTD, C-terminal domain.

Given the similarity of PICK1 to other endocytic accessory proteins with respect to AP2 binding, we investigated whether PICK1 also binds dynamin. Three dynamin genes exist in mammalian genomes, encoding three closely related proteins that are 80% identical, but with distinct patterns of expression (Ferguson and De Camilli, 2012). In co-IPs from cultured neurons, all three dynamins showed an interaction with PICK1 (Fig. 4 A). Furthermore, purified HA-tagged dynamin 2 bound directly to GST-PICK1 (Fig. 4 B). For our experiments that required recombinant dynamin, we used dynamin 2 because it is ubiquitously expressed and is well characterized. Dynamin is composed of distinct domains that support its function as a membrane-remodeling GTPase: a GTPase domain, a middle domain (MID), a pleckstrin homology domain (PH), a GTPase effector domain (GED), and a proline-rich domain (PRD; Fig. 4 C; Ferguson and De Camilli, 2012; Antonny et al., 2016). To investigate which domain interacts with PICK1, we performed GFP-trap pulldowns from HEK293 cells expressing PICK1 and GFP-tagged domains of dynamin 2. PICK1 showed a robust interaction with full-length dynamin 2 and specifically bound the isolated GTPase domain, with no detectable interaction with the other domains (Fig. 4 C). We used a similar approach to analyze the region of PICK1 that binds dynamin. PICK1 does not contain an SH3 domain or any other known dynamin-binding site but comprises a PDZ domain, a BAR domain, and an unstructured C-terminal domain (CTD) (Fig. 4 D). We performed GFP-trap pulldowns from HEK293 cells expressing dynamin 2 and GFP-tagged PICK1 truncations. As well as interacting with WT PICK1, dynamin showed a specific interaction with the BAR domain (Fig. 4 D). To investigate whether the DDAA mutation affects the PICK1 interaction with dynamin as well as AP2, we performed GST-PICK1 pulldowns from neuronal lysate. Interestingly, there was no significant difference in endogenous dynamin binding to GST-DDAA-PICK1 compared with GST-WT-PICK1 (Fig. S1 D), demonstrating that the dynamin–PICK1 interaction is not affected by AP2 binding, nor by the DDAA mutation per se.

To catalyze the fission of clathrin-coated vesicles from the plasma membrane during endocytosis, dynamin must polymerize into helical ring structures around the neck of the CCP (Ferguson and De Camilli, 2012; Antonny et al., 2016). Several dynamin-binding proteins such as Grb2 and SNX9 have been shown to promote dynamin polymerization, which can be analyzed in vitro using high-speed sedimentation assays (Barylko et al., 1998; Soulet et al., 2005). We applied this approach to determine whether such a role is performed by PICK1. His_6_-PICK1 caused a marked dose-dependent increase in dynamin polymerization in this assay (Fig. 4 E). As a control, we tested his_6_-NSF, which had no effect on dynamin polymerization (Fig. 4 F). We also tested PICK1 truncations, which showed that although neither the PICK1 C terminal nor the PDZ domain stimulated dynamin polymerization, his_6_-PICK1 CTDΔ was as effective as the full-length protein (Fig. 4 F). These experiments demonstrate that the PICK1 BAR domain binds dynamin directly to stimulate dynamin polymerization.

### PICK1 binding to AP2 and dynamin is transiently enhanced by NMDAR stimulation

**Figure 5.**
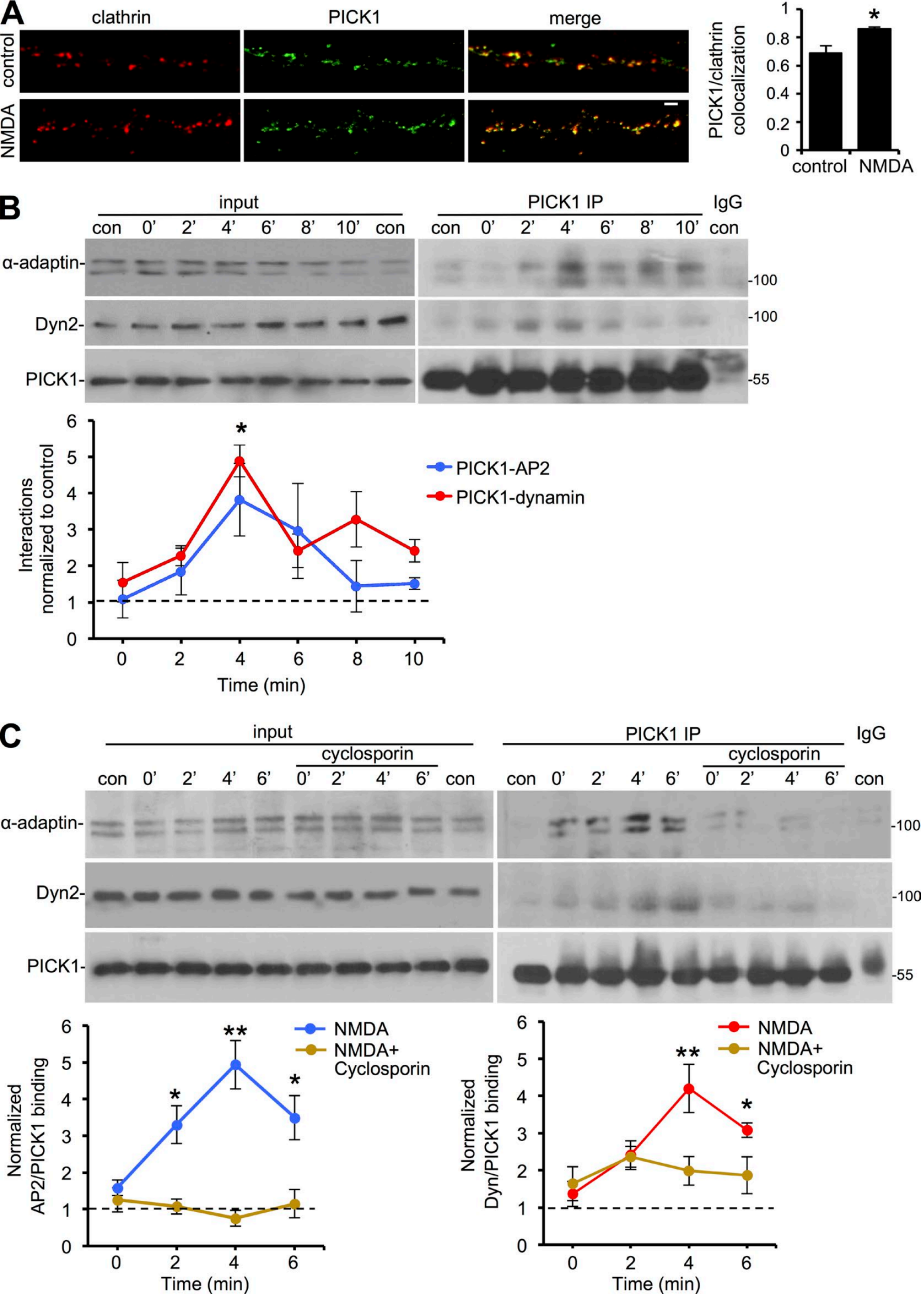
**PICK1–AP2 and PICK1–dynamin interactions are transiently enhanced after NMDAR stimulation by calcineurin activity.** (A) NMDAR stimulation causes an increase in PICK1–clathrin colocalization. Hippocampal neurons expressing dsRed-clathrin and PICK1 shRNA + GFP-WT-PICK1 were exposed to NMDA for 3 min and then returned to normal medium for 4 min. Representative STED images of dendrites are shown. Bar, 1 µm. Graph shows PICK1 colocalization with clathrin (Manders coefficient; *n* = 3 independent experiments [11–16 cells per condition in total]; *, P < 0.05; Student’s two-tailed *t* test). (B) NMDAR stimulation transiently increases PICK1 interaction with AP2 and dynamin. Neurons were exposed to NMDA and then returned to normal medium. Cell extracts were prepared for immunoprecipitation with anti-PICK1 antibodies at the indicated time points after the end of NMDA application. Proteins were detected by Western blotting. Graphs show quantification of PICK1–AP2 and PICK1–dynamin binding (*n* = 5 independent experiments; *, P < 0.05; one-way ANOVA followed by Tukey’s test). (C) Calcineurin activity is required for the NMDA-induced increase in PICK1–AP2 and PICK1–dynamin interactions. Neurons were treated as in B, except that cells were exposed to 10 µM cyclosporin A 1 h before NMDA application. Graphs show quantification of PICK1–AP2 and PICK1–dynamin binding (*n* = 8 independent experiments; *, P < 0.05; **, P < 0.01; one-way ANOVA followed by Tukey’s test; values are means ± SEMs).

We hypothesized that the novel interactions between PICK1 and the endocytic machinery would be a critical aspect of activity-dependent AMPAR endocytosis. As a first test for this hypothesis, we investigated whether NMDAR stimulation affected the localization of PICK1 to EZs in neuronal dendrites. Fig. 5 (A) demonstrates that NMDA caused a significant increase in colocalization between GFP-PICK1 and dsRed-clathrin, suggesting an NMDAR-dependent recruitment of PICK1 to EZs.

To investigate NMDAR-dependent changes in the binding of PICK1 to AP2 and dynamin, we performed co-IPs from lysates prepared from neuronal cultures at various time points after NMDAR stimulation. Both AP2 and dynamin interactions with PICK1 were transiently enhanced by NMDA application, with maximum binding at 4 min after NMDA washout (Fig. 5 B).

Several protein interactions involved in presynaptic vesicle endocytosis, including those involving BAR domain proteins, are enhanced by calcineurin-mediated dephosphorylation (Slepnev et al., 1998; Cousin and Robinson, 2001; Anggono et al., 2006). Given the well-established role for postsynaptic calcineurin in LTD (Mulkey et al., 1994; Beattie et al., 2000), we hypothesized that PICK1’s interaction with endocytic proteins might be regulated in a similar manner. Treating neuronal cultures with the specific calcineurin inhibitors 10 µM cyclosporin A (Fig. 5 C) or 1 µM FK506 (Fig. S4 A) blocked the NMDA-stimulated increase in PICK1–AP2 and PICK1–dynamin interactions, demonstrating that calcineurin activity is required for this effect. To confirm a role for calcineurin in regulating PICK1–AP2 binding, we analyzed the interaction between GFP-PICK1 and endogenous AP2 in HEK293 cells cotransfected with either constitutively active (CA) or phosphatase-dead (PD) calcineurin. CA calcineurin caused a significant increase in PICK1–AP2 binding, whereas PD calcineurin had no effect (Fig. S4 B), supporting a role for calcineurin activity in enhancing AP2 binding to PICK1. Taken together, these experiments demonstrate that NMDAR stimulation causes a transient, calcineurin-dependent increase in PICK1–AP2 and PICK1–dynamin interactions, providing a mechanism for transducing NMDAR stimulation to regulation of the endocytic machinery for AMPAR endocytosis.

### PICK1 is required for targeting AMPARs to EZs before its interaction with AP2

**Figure 6.**
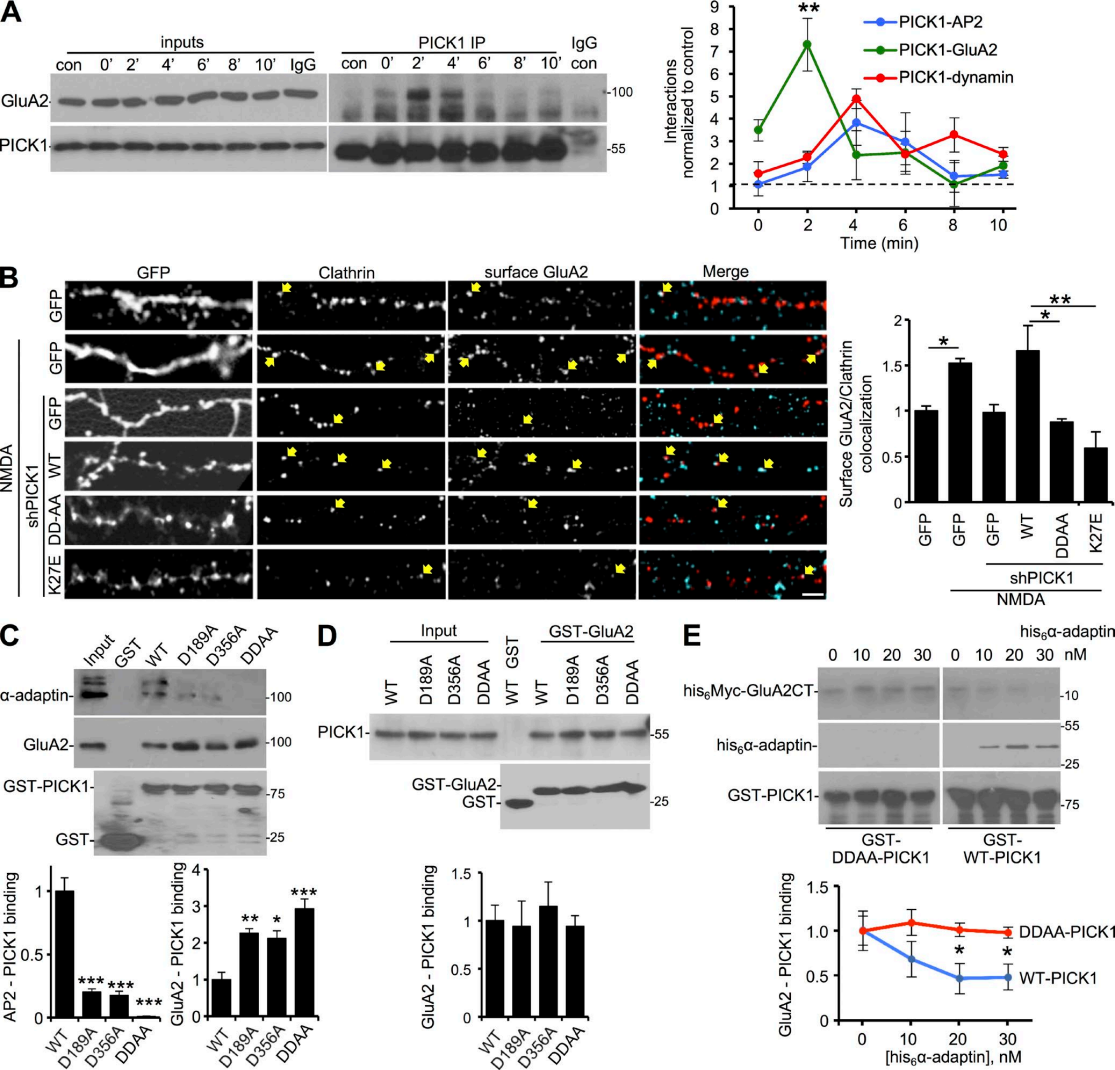
**PICK1 is required for clustering AMPARs at EZs via competitive binding of GluA2 and AP2.** (A) NMDAR stimulation transiently increases PICK1–GluA2 binding with a different time course compared with PICK1–AP2 and PICK1–dynamin. These data were acquired from the same experiments as the AP2 and dynamin data presented in Fig. 5 (B). Graph shows quantification of PICK1–GluA2 binding (*n* = 6 independent experiments; **, P < 0.01; one-way ANOVA followed by Tukey’s test). (B) PICK1–GluA2 and PICK1–AP2 interactions are required for the NMDAR-dependent clustering of GluA2 at EZs. Cultured hippocampal neurons expressing dsRed-clathrin and GFP, shPICK1 + sh-resistant GFP-WT-PICK1, or mutants, as indicated, were exposed to NMDA for 3 min and then returned to normal medium for 2 min. Live cells were labeled with GluA2 antibodies before fixation. Representative confocal images are shown. Arrows indicate overlapping puncta positive for GluA2 and clathrin. Bar, 5 µm. Graph shows surface GluA2 colocalization with clathrin (Manders coefficient; *n* = 3 independent experiments [18–27 cells per condition in total]; *, P < 0.05; **, P < 0.01; one-way ANOVA followed by Tukey’s test). (C) DDAA mutations increase PICK1 binding to GluA2 in neuronal lysates. GST, GST-PICK1, or mutants, as indicated, were immobilized on glutathione agarose and incubated with neuronal extracts. Proteins were detected by Western blotting. Graphs show quantification of PICK1 binding to AP2 and GluA2 (*n* = 4 independent experiments; *, P < 0.05; **, P < 0.01; ***, P < 0.001; one-way ANOVA followed by Tukey’s test). (D) DDAA mutations have no effect on PICK1–GluA2 binding in vitro using purified components. GST or GST-GluA2 was immobilized on glutathione agarose and incubated with purified his_6_-PICK1 or mutants as indicated. Graph shows quantification of PICK1–GluA2 binding (*n* = 4 independent experiments; one-way ANOVA followed by Tukey’s test). (E) AP2 directly competes with GluA2 for binding to PICK1. GST-WT-PICK1 or GST-DDAA-PICK1 were immobilized on glutathione agarose and incubated with the 20 nM his_6_-Myc-GluA2 C terminus and 0–30 nM his_6_ α-adaptin as indicated. Graph shows quantification of GST-PICK1 binding to the his_6_-Myc-GluA2 C terminus (*n* = 6 independent experiments; *, P < 0.05; two-way ANOVA followed by Bonferroni’s correction; values are means ± SEMs).

To further test our hypothesis that PICK1 regulates AMPAR endocytosis via interactions with AP2 and dynamin, we investigated the relative timing of PICK1 binding to GluA2 and to the endocytic machinery. Although an NMDAR-dependent increase in PICK1-GluA2 has been demonstrated previously (Iwakura et al., 2001), the temporal pattern of this interaction after stimulation is unknown. We found that PICK1–GluA2 interactions were also transiently increased after NMDAR stimulation, but showed a different time course, suggesting that PICK1 bound GluA2 before binding AP2 and dynamin (Fig. 6 A). AMPARs are thought to be internalized at EZs adjacent to the PSD (Lu et al., 2007; Opazo and Choquet, 2011); hence, their recruitment to these sites must be a critical first step in their endocytosis. The early peak in GluA2-PICK1 binding caused by NMDAR stimulation (Fig. 6 A) suggests a role for PICK1 in an early stage of AMPAR endocytosis, perhaps in the recruitment of GluA2-containing receptors to CCPs. To test this, we analyzed the colocalization of endogenous surface GluA2 with EZs. Under basal conditions, we detected a proportion of surface GluA2 puncta colocalizing with dsRed-clathrin clusters, which was significantly increased 2 min after NMDAR stimulation, strongly suggesting a recruitment of GluA2-containing AMPARs to CCPs (Fig. 6 B). At 2 min after stimulation, AMPARs had not yet internalized. This was blocked by PICK1 shRNA, indicating that PICK1 is required for this process. Furthermore, the NMDA-induced increase in surface GluA2 localization to EZs was rescued by sh-resistant WT-PICK1, but not by DDAA-PICK1, nor by K27E-PICK1, a mutation that blocks GluA2 binding (Citri et al., 2010). These results indicated that the PICK1–GluA2 and PICK1–AP2 interactions are required for clustering GluA2-containing AMPARs at EZs in response to NMDAR stimulation. Our data in Fig. 6 (A) suggest that the increase in PICK1–AP2 and PICK1–dynamin binding might be linked to the decrease in PICK1–GluA2 binding. To test this, we sought to determine whether PICK1 binding to AP2 influenced its interaction with GluA2 by performing pulldowns with GST-PICK1 mutants against neuronal lysates. Both the D189A and D356A mutations caused a significant increase in the interaction with GluA2 compared with WT-PICK1, and the DDAA mutation caused an even greater increase (Fig. 6 C), such that the increase in GluA2 binding mirrored the decrease in AP2 interaction seen with these mutants. Importantly, binding assays with purified proteins showed that these mutations had no effect on the direct interaction between his_6_-PICK1 and the GST-GluA2 C terminus (Fig. 6 D), strongly suggesting that the increase in GluA2 binding observed in neuronal lysates was caused by the reduction in AP2 binding. To test this directly, we performed competition assays using purified proteins. The his_6_ α-appendage domain specifically displaced the his_6_-GluA2 C terminus from GST-WT-PICK1 but not from GST-DDAA-PICK1 (Fig. 6 E), indicating that AP2 binding to PICK1 directly disrupts the interaction between GluA2 and PICK1. Collectively, these experiments suggest a mechanism in which NMDAR activation causes a rapid increase in PICK1–GluA2 interaction, which is required for clustering GluA2-containing AMPARs at EZs. PICK1 then associates with AP2 at CCPs and consequently dissociates from GluA2.

### PICK1–AP2 interaction does not involve PACSIN/syndapin

PACSIN/syndapin proteins are a family of BAR domain proteins that interact with dynamin and actin regulatory proteins and play a role in CME by linking actin polymerization with vesicle fission. It was reported previously that PICK1 associates with PACSIN/syndapin and that this interaction is involved in NMDA-stimulated internalization of GluA2-containing AMPARs (Anggono et al., 2013). A more recent study demonstrates that the PICK1–PACSIN/syndapin interaction is involved in AMPAR recycling and not endocytosis per se (Widagdo et al., 2016). Nevertheless, we explored whether PACSIN/syndapin might be involved in the interactions between PICK1 and the endocytic machinery and analyzed PACSIN/syndapin binding to DDAA-PICK1. We found that the AP2-binding mutations had no effect on the interaction with PACSIN/syndapin (Fig. S5 A), indicating that PACSIN does not bind the same site on PICK1 as AP2. We also investigated whether PACSIN/syndapin knockdown affected the NMDA-stimulated increases in PICK1–AP2 or PICK1–dynamin interactions. We used lentiviral vectors to express PACSIN/syndapin shRNA plus GFP or a GFP-tagged, sh-resistant PACSIN/syndapin, which were effective at depleting endogenous PACSIN/syndapin and expressing GFP-PACSIN/syndapin in hippocampal cultures, as reported previously (Anggono et al., 2013; Fig. S5 B). PACSIN/syndapin knockdown had no effect on the NMDA-stimulated increase in PICK1–AP2 binding (Fig. S5 C), indicating that these complexes are functionally distinct. However, depletion of PACSIN/syndapin blocked the NMDA-induced increase in PICK1–dynamin binding, which was rescued by molecular replacement with GFP-PACSIN/syndapin (Fig. S5 C). These results suggest a mechanism in which NMDAR stimulation causes an increase in PICK1 binding to AP2 in a PACSIN/syndapin-independent manner, and the same stimulus causes an increase in PICK1–dynamin binding that requires PACSIN/syndapin.

### PICK1 interaction with the endocytic machinery is required for basal and NMDA-induced AMPAR internalization

**Figure 7.**
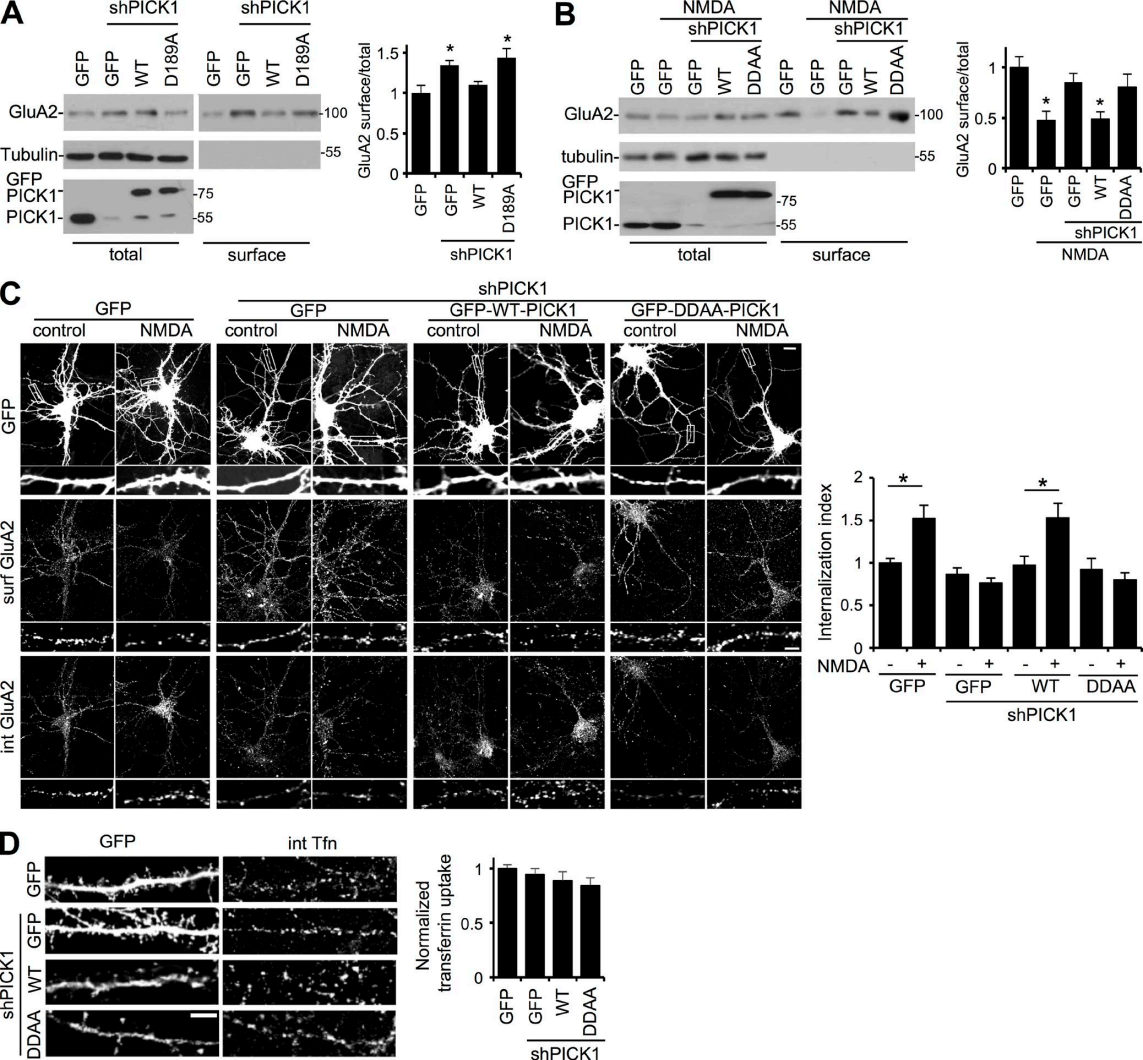
**PICK1–AP2 interaction is required for AMPAR endocytosis after NMDAR activation.** (A) PICK1–AP2 interaction is involved in constitutive AMPAR trafficking. Neurons infected with lentivirus expressing GFP, shPICK1 + GFP, shPICK1 + sh-resistant GFP-WT-PICK1, or shPICK1 + sh-resistant GFP-D189A-PICK1 were subjected to surface biotinylation. Representative blots show surface and 5% of the total GluA2 in lysates. (B) PICK1–AP2 interaction is required for NMDA-induced reduction in surface GluA2 containing AMPARs. Neurons infected with lentivirus expressing GFP, shPICK1 + GFP, shPICK1 + sh-resistant GFP-WT-PICK1, or shPICK1 + sh-resistant GFP-DDAA-PICK1 were exposed to NMDA for 3 min, returned to normal medium for 7 min, and subjected to surface biotinylation. Representative blots show surface and 5% of the total GluA2 in lysates. (A and B) Graph shows surface/total ratio (*n* = 5 independent experiments; *, P < 0.05; one-way ANOVA followed by Tukey’s test; values are means ± SEMs). (C) PICK1–AP2 interaction is required for NMDA-induced endocytosis of GluA2-containing AMPARs. Hippocampal neurons transfected with GFP, shPICK1 + GFP, shPICK1 + sh-resistant GFP-WT-PICK1, or shPICK1 + sh-resistant GFP-DDAA-PICK1 were surface labeled with GluA2 antibody before application of NMDA for 3 min. 7 min later, cells were fixed and stained for the internal and surface pools of GluA2 using different secondary antibodies. Bar, 5 µm. Graph shows internalization index (internalized/total; *n* = 5 independent experiments [35–45 cells per condition in total]; *, P < 0.05; one-way ANOVA followed by Tukey’s test; values are means ± SEMs). (D) PICK1 is not required for the endocytosis of transferrin receptors. Hippocampal neurons transfected with GFP, shPICK1 + GFP, shPICK1 + GFP-WT-PICK1, or shPICK1 + GFP-DDAA-PICK1 were surface labeled with Alexa Fluor 568–conjugated transferrin for 12 min. After acid wash, cells were fixed and imaged. Bar, 2 µm. Graph shows quantification of internalized transferrin (*n* = 3 independent experiments [12–14 cells per condition in total]).

To investigate the functional relevance of the PICK1–AP2 interaction in AMPAR trafficking, we initially performed surface biotinylation assays to analyze the internalization of endogenous GluA2-containing AMPARs. Under basal conditions, PICK1 knockdown caused an increase in surface GluA2, consistent with previous studies (Sossa et al., 2006; Citri et al., 2010; Rocca et al., 2013). Sh-resistant WT-PICK1 fully rescued surface GluA2 levels, but D189A-PICK1 did not (Fig. 7 A). This indicates that the interaction between PICK1 and AP2 is required for constitutive AMPAR endocytosis. We also used biotinylation to analyze surface GluA2 after NMDAR stimulation. In GFP-expressing control neurons, NMDA caused a significant decrease in surface GluA2, which was blocked by PICK1 shRNA (Fig. 7 B). Whereas sh-resistant WT-PICK1 rescued the phenotype, DDAA-PICK1 did not, indicating that PICK1–AP2 interactions are required for NMDA-stimulated AMPAR internalization. To further analyze NMDAR-dependent AMPAR endocytosis, we performed antibody-feeding immunocytochemistry, which specifically reports the internalization of AMPARs that originated on the cell surface. It has previously been suggested that at 5–10 min after NMDAR stimulation, the internalized pool detected by this method represents newly endocytosed AMPARs, whereas at later time points (around 15–20 min after stimulation), the internalized pool is strongly influenced by the extent of recycling from endosomal compartments back to the plasma membrane (Citri et al., 2010; Widagdo et al., 2016). 7 min after the NMDA stimulus, control neurons expressing GFP showed a robust increase in GluA2 internalization, which was blocked by PICK1 shRNA and rescued by sh-resistant WT-PICK1 but not by DDAA-PICK1 (Fig. 7 C).

To investigate whether an interaction between PICK1 and AP2 is involved in the endocytosis of a receptor that does not bind PICK1, we analyzed transferrin receptor internalization using transferrin uptake assays. Fig. 7 D demonstrates that neither PICK1 knockdown nor molecular replacement with DDAA-PICK1 affected transferrin uptake, indicating that PICK1 is not involved in transferrin receptor endocytosis. These results demonstrate that PICK1 regulates basal and NMDAR-dependent AMPAR endocytosis via its interaction with AP2 but does not regulate endocytosis of a receptor that does not interact with PICK1.

## Discussion

We define a new role for PICK1 as an endocytic accessory protein that interacts directly with the α-adaptin appendage domain of AP2 and with the dynamin GTPase domain in an activity-dependent manner to drive AMPAR internalization. Our results suggest a mechanism in neurons whereby NMDAR activation causes a rapid increase in PICK1–GluA2 binding, followed by a calcineurin-dependent increase in PICK1–AP2 binding, both of which are required for clustering GluA2-containing AMPARs at EZs. The increase in binding to AP2 causes PICK1 to dissociate from GluA2. The endogenous PICK1–dynamin interaction is also increased by NMDAR stimulation, requires calcineurin activity, and follows a similar time course as the PICK1–AP2. Our results from heterologous cells and in vitro experiments suggest that PICK1 localizes to CCPs in an AP2-dependent and dynamin-independent manner and can subsequently stimulate dynamin polymerization via direct interaction between the BAR domain and dynamin.

### AMPAR clustering at CCPs

It has been suggested that NMDAR-dependent internalization of synaptic AMPARs requires the dissociation of AMPARs from PSD-95 scaffolds in the PSD, allowing lateral diffusion away from the synapse and subsequent endocytosis at adjacent EZs (Opazo and Choquet, 2011). Our data demonstrate a requirement for PICK1–GluA2 and PICK1–AP2 binding for clustering GluA2 at EZs, yet also indicate that PICK1 binding to GluA2 and to AP2 is competitive. We therefore propose a mechanism in which the PICK1–AP2 interaction causes GluA2-containing AMPARs to cluster at EZs, where the high concentration of AP2 at CCPs favors PICK1–AP2 binding, causing PICK1 to dissociate from GluA2. Moreover, dissociation of PICK1 from the receptor cargo is necessary if PICK1 is to localize at the neck of the CCP to regulate dynamin polymerization. Our results from the dynamin TKO fibroblasts indicate that PICK1 is recruited to CCPs in an AP2-dependent manner. As well as colocalizing with clathrin, PICK1 colocalizes with endophilin-A2, which is a marker for the CCP neck. These results are consistent with a model in which PICK1 is initially recruited to endocytic sites via its interaction with AP2 in the clathrin coat and subsequently associates with the CCP neck. The presence of PICK1 at both the clathrin coat and neck regions of the CCP simultaneously could be caused by disruption of the late stages of endocytosis in these cells and hence reduced dynamics of the system.

The molecular basis for the competition between GluA2 and AP2 for binding to PICK1 is unclear because GluA2 binds the PDZ domain and AP2 binds two sites, one in the BAR domain and one immediately adjacent to the BAR domain. However, it has been suggested that GluA2 makes a second, non-PDZ contact with PICK1 between a membrane-proximal region (amino acids 846–851) of the GluA2 C-terminal tail and the PICK1 BAR domain, which has a significant influence on PICK1 binding to GluA2 (Hanley et al., 2002). This observation provides a potential mechanistic explanation for the competition; AP2 binding to the PICK1 BAR domain might disrupt the interaction with this membrane-proximal region of GluA2.

An important aspect of CME is the temporal characteristics of the numerous protein–protein interactions involved and the order in which they take place (Schmid and McMahon, 2007). In the NMDAR-dependent mechanism described herein, a mechanism to define the sequence of events such that PICK1 binds GluA2 before it binds AP2 would be essential for the targeting of AMPARs to EZs by PICK1. We show that PICK1–GluA2 binding is enhanced markedly earlier than interactions between PICK1 and the endocytic machinery. This is consistent with the competition between GluA2 and AP2 for binding to PICK1. An early interaction with GluA2 will inhibit the interaction with AP2, and a subsequent increase in interaction with AP2 will disrupt GluA2 binding. It also suggests that the signaling mechanisms involved in increasing PICK1–GluA2 binding have a different time course compared with the calcineurin-dependent events that underlie the increase in PICK1–AP2 and PICK1–dynamin interactions. Our data also suggest that the PICK1–AP2 and PICK1–dynamin interactions occur at a similar time point after NMDAR stimulation. However, we acknowledge that our biochemical approach does not yield high temporal resolution of protein interactions. Although our results indicate that the binding of AP2 to PICK1 does not influence the PICK1–dynamin interaction, we hypothesize that PICK1 binds dynamin at a later time point after stimulation than AP2 because dynamin polymerization occurs at a late stage in the lifetime of a CCP (Ferguson and De Camilli, 2012; Daumke et al., 2014). This hypothesis is further supported by our results suggesting a requirement for PICK1–AP2 interactions in the clustering of AMPARs at EZs, which presumably represents an early stage in the formation of an AMPAR-containing CCP. Further support for a mechanism in which PICK1 binding to AP2 and to dynamin are independent events comes from our experiments involving PACSIN/syndapin. PICK1–AP2 binding is unaffected by PACSIN/syndapin, whereas the NMDA-induced increase in PICK1–dynamin interactions appears to require this BAR and SH3 domain–containing protein, which is itself known to bind dynamin (Anggono et al., 2006). The role of PACSIN in the PICK1–dynamin interaction is unclear. We show that PICK1 binds dynamin directly, so it is unlikely that the PICK1–dynamin interaction is physically mediated by PACSIN/syndapin. Perhaps PICK1 and PACSIN/syndapin play a synergistic role in the regulation of dynamin function in response to NMDAR stimulation. In addition, an interaction between PICK1 and the multi-PDZ domain protein GRIP has also been implicated in AMPAR endocytosis (Lu and Ziff, 2005). In the model proposed in this previous study, PICK1 binds GRIP before it binds GluA2. Because we show that PICK1–GluA2 binding occurs before PICK1–AP2 in the response to NMDAR stimulation, we believe that it is very unlikely that GRIP is involved in the interaction of PICK1 with AP2 and dynamin. Additional experiments will be required to precisely define the timing and hence the orchestration of all these protein–protein interactions in AMPAR endocytosis.

Similar to numerous other endocytic cargoes, GluA2 itself and the AMPAR accessory protein stargazin both bind directly to the μ2 subunit of AP2, and these interactions are necessary for NMDAR-dependent AMPAR endocytosis (Lee et al., 2002; Kastning et al., 2007; Matsuda et al., 2013). This leads to the question of why PICK1 is necessary for targeting AMPARs to CCPs. A possible explanation lies in the structure of α-adaptin. The appendage domain that binds endocytic accessory proteins is found at the end of a long linker region with little tertiary structure, which can sample a large area but forms a compact structure that pulls binding proteins close into the core AP2 complex (Praefcke et al., 2004). Because PICK1 binds GluA2 before AP2 in response to NMDAR activation, the α-appendage domain first interacts with PICK1 when PICK1 is bound to GluA2. Therefore, we propose that PICK1 plays a critical role in the efficient recruitment of GluA2 to CCPs because the α-appendage can reach farther to contact PICK1 compared with the μ2 subunit contacting GluA2 or stargazin.

CME requires localized regulation of the actin cytoskeleton to generate mechanical forces that contribute to the complex alterations in membrane geometry that underlie CCP formation and scission. The Arp2/3 complex is the major catalyst for the formation of branched actin networks that mediate such changes in membrane geometry (Campellone and Welch, 2010). It has been suggested previously that the Arp2/3 activator N-WASP is recruited to CCPs at a similar time point as dynamin to provide a burst of actin polymerization at the neck of the CCP to drive the late stages of invagination and vesicle scission (Taylor et al., 2011). PICK1 binds and inhibits the Arp2/3 complex, and this process is required for NMDA-stimulated AMPAR internalization (Rocca et al., 2008; Madasu et al., 2015), suggesting that it plays a role in Arp2/3 regulation during CME of AMPARs. Because dendritic spines are particularly rich in F-actin, we propose that Arp2/3 inhibition is required to reduce the local density of actin filaments around the developing CCP before the recruitment of N-WASP, thus providing sufficient dynamic range of actin polymerization to generate the necessary mechanical forces for vesicle fission. Future work will precisely define the role of PICK1-mediated Arp2/3 inhibition in AMPAR endocytosis.

### Regulation of dynamin

Our results strongly suggest that a second role for PICK1 is to promote dynamin polymerization at the neck of the CCP. PICK1 shares functional features with other endocytic BAR domain proteins (e.g., amphiphysin and SNX9) that are recruited to CCPs by AP2 and regulate dynamin for vesicle fission. A notable difference, however, is that amphiphysin and SNX9 (and numerous other dynamin-interacting proteins) bind the dynamin PRD via an SH3 domain (Ferguson and De Camilli, 2012; Antonny et al., 2016). PICK1 does not contain an SH3 domain; instead, the BAR domain binds directly to the dynamin GTPase domain. Dynamin helical polymers are thought to assemble in a GTP-dependent manner via GTPase domain–GTPase domain interactions between dimers in adjacent rungs of the helix (Ferguson and De Camilli, 2012); hence, our results suggest that PICK1 modulates dynamin polymerization by influencing these interactions between GTPase domains. The relevance of this difference between PICK1 and SH3 domain–containing proteins is unclear but is likely to reflect distinct modes of dynamin regulation, despite our observation that PICK1 promotes dynamin self-assembly in a similar manner to SNX9 (Soulet et al., 2005).

### Synaptic plasticity

Previous studies from other laboratories have suggested that the function of PICK1 in synaptic plasticity is not to promote GluA2 endocytosis but instead to restrict their recycling from endosomal compartments (Lin and Huganir, 2007; Citri et al., 2010; Widagdo et al., 2016). Although our results do not exclude a role for PICK1 in regulating endosomal recycling, they strongly support its role in endocytosis. The reason for the lack of effect on AMPAR endocytosis reported in these previous studies is unclear. However, one consistent difference between these and our experiments is that our NMDA stimulus lasted for 3 min compared with 5 min in the other studies. Perhaps the extended stimulation time causes PICK1 to bypass the endocytic machinery and instead bind preferentially to proteins involved in endosomal recycling. Because LTD involves critical AMPAR sorting steps in the endosomal system as well as an increase in AMPAR endocytosis (Lee et al., 2004; Fernández-Monreal et al., 2012), we propose that PICK1 is involved in the control of both AMPAR endocytosis and recycling via distinct patterns of protein interactions.

Calcineurin has a well-established role in the expression of LTD; however, few substrates apart from GluA1 and PSD-95 have been identified that play a role in postsynaptic plasticity (Banke et al., 2000; Beattie et al., 2000; Kim et al., 2007). Our results indicate that the NMDA-dependent increases in both the PICK1–AP2 and PICK1–dynamin complexes require calcineurin activity. It is interesting to note that a previous study reported an interaction between PICK1 and the regulatory subunit of calcineurin (Iida et al., 2008), suggesting that PICK1 might function as a scaffold to bring calcineurin close to substrates or indeed that PICK1 itself is a calcineurin substrate.

Activity-dependent GluA2 endocytosis is a central mechanism for LTD that underlies specific kinds of learning (Griffiths et al., 2008). It is more recently emerging as a key mechanism in memory decay, in which AMPARs are removed from previously potentiated synapses by GluA2-dependent endocytosis (Hardt et al., 2014; Migues et al., 2016). In addition, synaptic plasticity involving GluA2 internalization has been suggested to play a role in neurological disorders, such as brain ischemia, traumatic brain injury, and Alzheimer’s disease (Hsieh et al., 2006; Bell et al., 2009; Dixon et al., 2009; Chen et al., 2014). Therefore, we propose that the functional interactions between PICK1 and the endocytic machinery are critical components of these forms of synaptic plasticity and might represent novel targets for therapeutic intervention in disease states.

In conclusion, we have identified a novel role for PICK1 as an AP2- and dynamin-interacting endocytic accessory protein. To our knowledge, PICK1 is the only protein with a PDZ domain that associates with AP2 appendage domains. It is therefore uniquely equipped to link plasma membrane receptor cargo containing cognate PDZ ligands to the core endocytic machinery. Furthermore, the Ca^2+^-binding properties of PICK1 (Hanley and Henley, 2005) provide a mechanism for transducing Ca^2+^ signals to endocytic cargo selection. In addition to the AMPAR subunit GluA2, the PICK1 PDZ domain binds glutamate transporters, Eph receptors, metabotropic glutamate receptors, and acid-sensing ion channels, among others (Li et al., 2016). Although it is unknown whether PICK1 has a similar role in the trafficking of these other proteins, it may be a common mechanism for efficient cargo selection and endocytosis of specific plasma membrane proteins in response to Ca^2+^ signals.

## Materials and methods

### Plasmids and oligonucleotide primers

His_6_ proteins were expressed from pET28 (Novagen); GST fusions from pGEX4T-1 (Pharmacia). GST-adaptin and his_6_-adaptin constructs were donated by V. Haucke (Max Delbruck Center for Molecular Medicine, Berlin, Germany). For HEK293 cell experiments, full-length PICK1 was expressed from pcDNA3.1, GFP-tagged truncations of PICK1 and dynamin were expressed from pEGFP-C1 (Clontech), and HA-tagged dynamin 2 was expressed from pcDNA3 (Addgene). Endophilin A2–EGFP, mRFP-clathrin, and EEA1-mRFP were donated by P. De Camilli (Department of Neuroscience, Yale University School of Medicine, New Haven, CT). mRFP-rab5, mRFP-Rab7, mCherry-Rab11a, and LAMP1-RFP were purchased from Addgene. pGEX-amphiphysin SH3 domain was donated by H. McMahon (Medical Research Council Laboratory of Molecular Biology, Cambridge, England, UK). Myc-PACSIN was donated by I. Perez-Otano (Instituto de Neurociencias, Consejo Superior de Investigaciones Científicas, Universidad Miguel Hernández, Alicante, Spain). Flag CA calcineurin and Flag PD calcineurin in pCMV-Flag were donated by P. Marin (Centre National de la Recherche Scientifique, Institut de Génomique Fonctionnelle, Montpellier, France). PICK1 shRNA and GFP-tagged rescue/molecular replacement constructs were expressed from a modified FUGW (Citri et al., 2010; Antoniou et al., 2014). The PICK1 shRNA was encoded by the following DNA sequence: CTATGAGTACCGCCTTATCCT. PACSIN/syndapin shRNA and GFP-tagged rescue/molecular replacement constructs were donated by R. Huganir (Solomon H. Snyder Department of Neuroscience, The Johns Hopkins University School of Medicine, Baltimore, MD). The PACSIN/syndapin shRNA was encoded by the DNA sequence 5′-GCGCCAGCTCATCGAGAAA-3′. 

For lentiviral production, HEK293TN cells were transfected with both FUGW and helper vectors pMDLg-pRRE, pRSV-Rev, and pVSV-G using polyethylenimine (Sigma Aldrich). 48 h after transfection, supernatant was harvested and concentrated by ultracentrifugation. Particles were aliquoted, titered, and stored at −80°C.

DNA mutagenesis primers were as follows (mutations are underlined): D189A, 5′-CGGGCTTTTGGGGCCGTGTTCTCTGTG-3′ and 5′-CACAGAGAACACGGCCCCAAAAGCCCG-3′; D356A, 5′-CTGCGGGACGCCGACGTCTTCCCCATC-3′ and 5′-GATGGGGAAGACGTCGGCGTCCCGCAG-3′.

### Antibodies

The antibodies used were as follows: PICK1 (mouse, 75–040; Neuromab; rabbit, ab3420; Abcam; goat, sc-9539; Santa Cruz Biotechnology); α-adaptin (mouse, 610502; Becton Dickinson); GST (mouse, 71097–3; Novagen); Arp2/3 complex (Arp3; mouse, A5979’Sigma Aldrich); Dynamin 1, Dynamin 2, and Dynamin 3 (rabbit, PA1-660, PA5-19800, and PA1-662; PIERCE); GluA2 (rabbit, 182 103; Synaptic Systems; mouse, 556341; Becton Dickinson); Homer (rabbit, 160 003; Synaptic Systems); β-actin (mouse, A2228; Sigma Aldrich); tubulin (mouse, T9026; Sigma Aldrich); GFP (mouse, 75–131; Neuromab); dsRed/mCherry (goat, AB0081-200; Sicgen); PACSIN (rabbit, 193 003; Synaptic Systems); Flag (mouse, F1804; Sigma Aldrich); myc (mouse, s-40; Santa Cruz Biotechnology); Alexa Fluor 568–conjugated transferin (123365; Molecular Probes); EEA1 (rabbit, C45B10; Cell Signaling Technologies); Rab11 (rabbit, D4F5; Cell Signaling Technologies); and GM130 (mouse, 610822; BD Bioscience).

### Buffers

The lysis buffer used is as follows: 20 mM Tris-HCl, pH 7.5, 150 mM NaCl, 0.5% Triton X-100, and protease inhibitor cocktail (Roche). The lysis buffer for biotinylations used was 20 mM Tris-HCl, pH 7.5, 150 mM NaCl, 1% Triton X-100, 0.1% SDS, 1 mM EDTA, and protease inhibitor cocktail (Roche). The direct binding buffer used was 20 mM Tris-HCl, 150 mM NaCl, 5 mM imidazole, 2 mM MgCl_2_, 1% Triton X-100, and 1 mM DTT. The dynamin purification buffer used was 20 mM Hepes, pH 7.4, 150 mM NaCl, 0.1% Triton X-100, 1 mM DTT, and protease inhibitor cocktail (Roche). The dynamin elution buffer used is as follows: 20 mM Pipes, pH 6.2, 1.2 M NaCl, 1 mM DTT, and protease inhibitor cocktail (Roche). The dynamin polymerization buffer used was 10 mM Hepes, pH 7.2, 2 mM MgCl_2_, and 150 mM KCl. The lipid binding buffer used was 20 mM Hepes, pH 7.4, 150 mM NaCl, and 1 mM DTT.

### Preparation of recombinant proteins

His_6_ and GST fusions were expressed and purified as described previously (Rocca et al., 2008). In brief, BL21 bacteria were transformed with pGEX or pET plasmids carrying relevant DNA inserts. Cultures from single colonies were harvested and lysed in buffer containing 1% Triton X-100 with sonication, followed by centrifugation to clear insoluble material. GST fusions were immobilized by incubating lysates with glutathione agarose (Sigma Aldrich) at 4°C. For his_6_ fusions, lysates containing 20 mM imidazole were incubated with Ni-NTA agarose (Qiagen) at 4°C, followed by extensive washing in buffer containing 40 mM imidazole. Proteins were eluted in 0.5-ml fractions in buffer containing 200 mM imidazole.

### Co-IPs and GFP-trap

Co-IPs were performed as described previously (Rocca et al., 2008). In brief, extracts of cortical neuronal cultures were prepared in lysis buffer and subsequently incubated with 5 µg anti-PICK1 or control IgG antibodies plus protein G—Sepharose (GE Healthcare). For GFP-trap, extracts of transfected HEK293 cells were prepared in lysis buffer and incubated with 10 µl GFP-trap agarose beads (ChromoTek). Beads were washed and proteins were detected by Western blotting.

### GST pulldown assays

Pulldown assays were conducted as described previously (Rocca et al., 2008). In brief, GST or GST-tagged proteins were immobilized on glutathione agarose beads in lysis buffer at 4°C. After washing, beads were incubated at 4°C with cell extracts prepared in lysis buffer or purified proteins in direct binding buffer. Beads were washed and proteins were detected by Western blotting.

### Dynamin TKO cells: Induction, transfection, and imaging

Loss of dynamin was induced in fibroblasts carrying floxed alleles for all three mammalian dynamins (Line A) using 4-hydroxytamoxifen (OHT; Sigma Aldrich) as described previously (Ferguson et al., 2009). In brief, Line A cells were treated for 2 × 24 h with 2 µM OHT in the DMEM medium containing 10% fetal bovine serum, causing dynamin depletion at 4–5 d after treatment start. Loss of dynamin was verified by Western blotting using the pan-dynamin antibody. Cells were transfected after 4 d of treatment by electroporation (P4 Primary Cell 4D-Nucleofector X Kit L; Amaxa; Lonza) according to the manufacturer’s protocol. Live cells were imaged up to 45 h after transfection on an Ultraview spinning-disk confocal setup (Perkin Elmer) consisting of an inverted microscope (Ti-E Eclipse; Nikon) equipped with Perfect Focus, a 60× CFI Plan Apo VC Nikon objective, and a 14-bit electron-multiplied charge-coupled device camera (C9100; Hamamatsu). Alternatively, fibroblasts were fixed in 4% PFA for 1 h, excess PFA was neutralized by a 20-min wash in 50-mM NH_4_Cl in PBS, and the coverslip was mounted to a microscope slide with Mowiol 4–88 (Sigma Aldrich). Slides were imaged with a Leica LSM 800 (Zeiss) setup.

### Dynamin polymerization assays

HEK 293 cells were transfected with pcDNA3 HA–dynamin 2 using jetPEI transfection reagent (Polyplus). After 3 d, cells were lysed in dynamin purification buffer and centrifuged at 14,000 rpm for 20 min. The supernatant was incubated for 1 h with the GST-SH3 domain of amphiphysin 2 immobilized on glutathione agarose beads, and purified dynamin was eluted with dynamin elution buffer.

0.2 µM purified dynamin was incubated in sedimentation buffer at room temperature for 30 min with increasing amounts of His-tagged proteins. The reaction mix was centrifuged at 4°C for 15 min at 20,000 *g*, and the presence of dynamin in the pellet and in the supernatant was analyzed by Western blotting.

### Lipid binding and F-actin binding assays

These were performed as described previously (Rocca et al., 2008). In brief, for lipid binding, brain lipid extracts (Folch extract I; Sigma Aldrich) were resuspended in 20 mM Hepes, pH 7.4, 150 mM NaCl, and 1 mM DTT. 2.5 µM his_6_-PICK1 was then incubated with 1 mg/ml lipid extract for 15 min at 37°C followed by centrifugation at 35,000 *g* for 15 min. Supernatant and pellet protein samples were resolved by SDS-PAGE and visualized by Coomassie staining. For F-actin binding, 100 nM his_6_-PICK1 was added to 5 µM G-actin in 50 mM KCl, 10 mM Tris, pH 7.5, 2 mM MgCl_2_, 0.2 mM ATP, and 0.2 mM DTT; incubated at room temperature for 1 h to allow F-actin assembly; and then centrifuged for 20 min at 250,000 *g*. Pellets and supernatants were analyzed by SDS-PAGE and Coomassie staining.

### Quantification of SDS-PAGE and Western blots

Western blot films were scanned and analyzed using ImageJ software (National Institutes of Health). Error bars are SEMs, and statistical tests were performed on the data using GraphPad Prism.

### Primary neuronal culture

Rat embryonic neuronal cultures were prepared from E18 Wistar rats using standard procedures. The culture medium was Neurobasal medium (Gibco) supplemented with B27 (Gibco) and 2 mM glutamine. Neurons were transfected with plasmid DNA at day in vitro (DIV) 10–13 (unless otherwise stated) using Lipofectamine 2000 (Invitrogen) and used for experiments 4–6 d later. For NMDAR stimulation, neurons were incubated with 1 µM tetrodotoxin (TTX) for 10–30 min and then stimulated with 50 µM NMDA (Tocris), 20 µM glycine, and 0.5 µM TTX for 3 min in Hepes-buffered saline (25 mM Hepes, pH 7.4, 140 mM NaCl, 5 mM KCl, 1.8 mM NaCl, 0.8 mM MgCl_2_, and 10 mM glucose).

### Surface biotinylation assays

Primary neuronal cultures were infected with lentiviruses at DIV 3–4 for 6 h before being returned to conditioned media. Cultures were then used for experiments at DIV 15–19. Biotinylations were performed as described previously (Hanley and Henley, 2005). In brief, neurons were stimulated if appropriate and then chilled on ice, washed in ice-cold PBS, and incubated with 0.25 mg/ml EZLink NHS-SS-Biotin (Pierce) in PBS for 10 min on ice. After washing three times in PBS plus 1 mg/ml BSA and three times in PBS, cells were lysed in a 20 mM Tris-HCl, pH 7.5, 150 mM NaCl, 1% Triton X-100, 0.1% SDS, 1 mM EDTA, and protease inhibitor cocktail (Roche). After centrifugation, lysate was incubated with streptavidin agarose beads for 3 h at 4°C and washed four times in the same buffer; bound protein was detected by Western blotting.

### Immunocytochemistry and image analysis

For surface staining of AMPARs, live hippocampal neurons (DIV 15–17) transfected with dsRed-clathrin and treated with 50 µM NMDA for 3 min at 37°C were surface labeled with anti-GluA2 antibodies in Hepes-buffered saline (HBS; 25 mM Hepes, pH 7.4, 140 mM NaCl, 5 mM KCl, 1.8 mM NaCl, 0.8 mM MgCl_2_, 10 mM glucose) for 20 min at room temperature. Neurons were fixed in 4% PFA + 4% sucrose and stained with anti–mouse Alexa Fluor 647 secondaries. For whole-cell staining, neurons were fixed for 12 min in 4% PFA + 4% sucrose; permeabilized with 0.1% Triton X-100; and incubated with anti-Homer, anti-GFP, or anti-mCherry/dsRed antibodies. Images were acquired on a Leica SP5II confocal microscope with a 63× NA 1.4 or a 40× NA 1.25 oil immersion objective using Fluoromount F4680 (Sigma Aldrich) at room temperature. Colocalization was analyzed by thresholding images (Otsu’s method) and applying the Coloc 2 plugin in Fiji. Three randomly selected 30-µM dendritic regions were analyzed per neuron.

For antibody-feeding experiments, live hippocampal neurons (DIV 15–17) were surface labeled with anti-GluA2 antibodies for 20 min at room temperature in HBS (1 µM TTX). Neurons were then washed in HBS and treated with 50 µM NMDA plus 20 µM glycine for 3 min at 37°C, followed by a 7-min chase without drugs. Neurons were fixed for 5 min with 4% PFA + 4% sucrose and stained with anti–mouse Alexa Fluor 647 secondaries. After a 12-min fixation in PFA, cells were permeabilized and stained with anti–mouse Alexa Fluor 568 secondaries. Images were acquired on a Leica SP5II confocal microscope with a 63× NA 1.4 or a 40× NA 1.25 oil immersion objective using Fluoromount F4680 (Sigma Aldrich) at room temperature and analyzed using ImageJ software. The internalization index was calculated by dividing the value corresponding to internalized staining by the value corresponding to total staining (internalized + surface). The GFP signal was used as a mask, and the mean fluorescence intensity was measured within this area. For transferrin uptake assays, neurons were incubated with 50 µg/ml Alexa Fluor 568–conjugated transferrin (Molecular Probes) for 12 min at 37°C. Cells were incubated on ice for 2 min, washed in 0.2 M acetic acid and 0.5 M NaCl for 3 min, and then fixed in 4% PFA + 4% sucrose for 12 min.

### STED imaging

Neurons were transfected with dsRed-clathrin and shPICK1 + GFP-PICK1 and fixed and labeled with anti-dsRed/mCherry and anti-GFP antibodies. Images were acquired with a 100× NA 1.4 oil immersion objective using Prolong Gold (Invitrogen) at 37°C on a Leica SP8 gated STED microscope equipped with two depletion lasers (592 and 660 nm). This enables super-resolution imaging to ∼70 nm xy. Alexa Fluor 488–labeled proteins were excited with the 488-nm-wavelength laser and depleted with a 592-nm STED laser, whereas Alexa Fluor 568–labeled proteins were excited with the 561-nm-wavelength laser and depleted with a 660-nm STED laser. Scan frequency was set at 80 MHz. All images were processed using a Gaussian blur filtering function in Fiji.

### Statistical analysis

All quantified Western blot and imaging data are presented as the mean of at least three independent experiments. Means and standard errors were calculated using GraphPad Prism, followed by a two-tailed *t* test or a one-way ANOVA and post hoc Tukey’s test to determine statistical significance. Data distribution was assumed to be normal, but this was not formally tested. For all statistical tests, P < 0.05 was considered significant and is indicated by an asterisk.

### Online supplemental material

Fig. S1 shows that DDAA mutation has no effect on PICK1 interactions with lipid, Arp2/3, F-actin, or dynamin or on PICK1 dimerization. Fig. S2 shows localization of DDAA-PICK1 in neurons. Fig. S3 shows localization of PICK1 DDAA to endomembrane compartments. Fig. S4 provides additional evidence of a role for calcineurin in regulating PICK1–AP2 and PICK1–dynamin interactions. Fig. S5 shows that PICK1–dynamin interaction, but not PICK1–AP2 interaction, involves PACSIN/syndapin. Video 1 shows WT-PICK1 colocalized with endophilin-A2. Video 2 shows WT-PICK1 colocalized with clathrin. Video 3 shows that DDAA-PICK1 does not associate with clathrin but localizes to a juxtanuclear area.

## Supplementary Material

Supplemental Material (PDF)

Video 1

Video 2

Video 3
