# Supplementary material for: PICK1 regulates AMPA receptor endocytosis via direct interactions with AP2 α-appendage and dynamin
Source: J Cell Biol. 2017 Oct 2;216(10):3323–38. doi: 10.1083/jcb.201701034 (PMC5626541; doi:10.1083/jcb.201701034)
Supplement: Supplemental Material (PDF) [file JCB_201701034_sm.pdf]

Fiuza et al., https://doi.org/10.1083/jcb.201701034

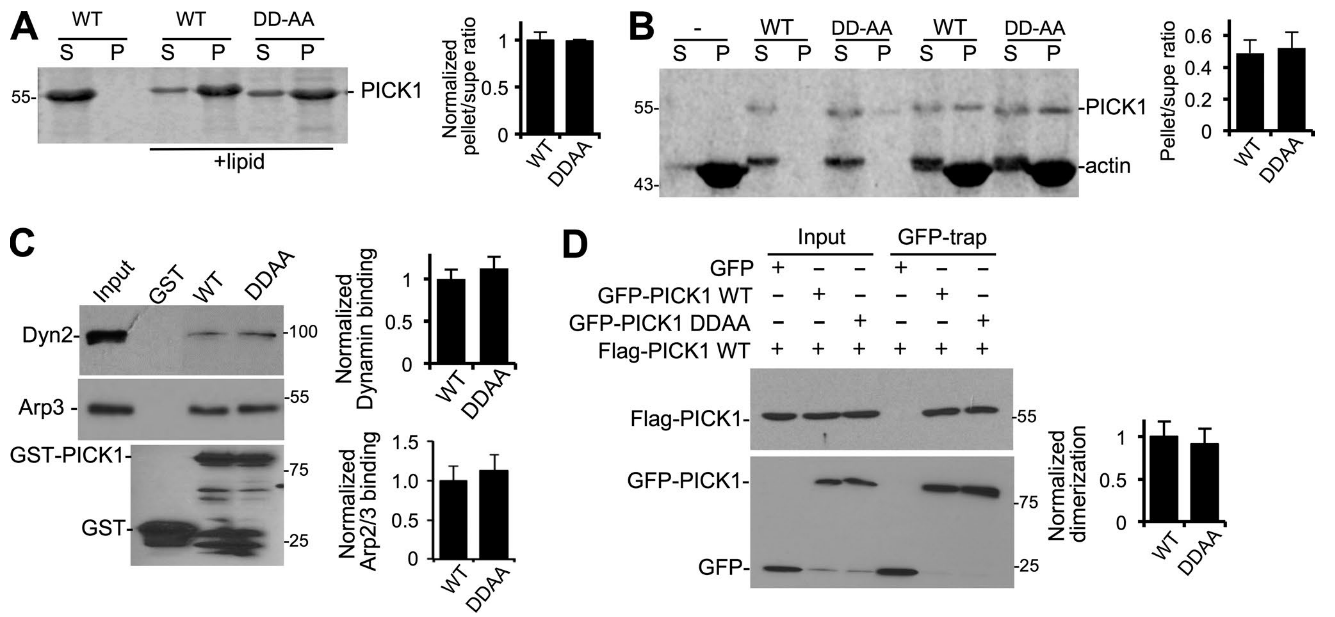

Figure S1. **DDAA mutation has no effect on PICK1 interactions with lipid, Arp2/3, F-actin, or dynamin or on PICK1 dimerization.** Related to Figs. 2 and 4. (A) DDAA mutation does not affect PICK1 interaction with phospholipids. Purified his_6-WT-PICK1 and his_6-DDAA-PICK1 were incubated with or without brain lipid extract (Folch extract) and subjected to ultracentrifugation to pellet the liposomes. Protein content of the pellet (P) and supernatant (S) was analyzed by SDS-PAGE and Coomassie staining. Graph shows quantification of PICK1 binding to lipids (pellet/supernatant ratio; *n* = 4; two-tailed *t* test). (B) DDAA mutation does not affect PICK1 interaction with F-actin. Purified and clarified his_6-WT-PICK1 and his_6-DDAA-PICK1 were incubated with or without 5 µM F-actin and subjected to ultracentrifugation to pellet actin filaments and associated proteins. Protein content of the pellet (P) and supernatant (S) was analyzed by Coomassie staining. Graph shows quantification of PICK1 binding to F-actin (pellet/supernatant ratio; *n* = 4; two-tailed *t* test). (C) DDAA mutation does not affect PICK1 interaction with Arp2/3 complex or with dynamin. GST, GST-WT-PICK1, or GST-DDAA-PICK1 were immobilized on glutathione agarose and incubated with neuronal extracts. Bound protein was detected by Western blotting. Graphs show quantification of PICK1 binding to Arp2/3 (*n* = 4) and to dynamin (*n* = 8; two-tailed *t* test). (D) DDAA mutation does not affect PICK1 dimerization. Lysates of HEK293 cells transfected with GFP, GFP-WT-PICK1, GFP-DDAA-PICK1, and flag-WT-PICK1, as shown in the figure, were incubated with GFP-trap beads, and bound proteins were detected by Western blotting. Graph shows quantification of flag-PICK1 binding to GFP-PICK1 (*n* = 4; two-tailed *t* test; values represent means ± SEMs).

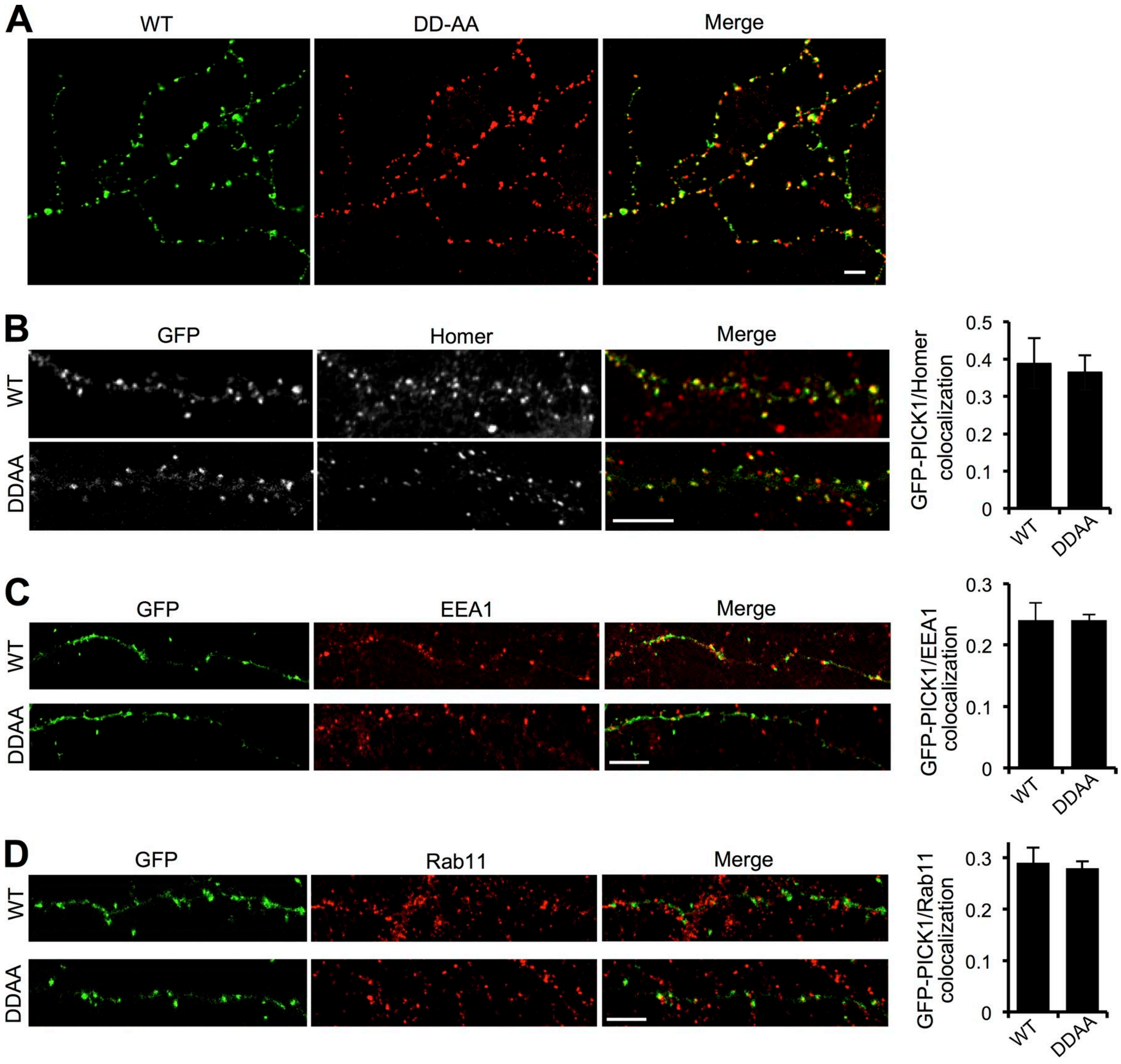

Figure S2. **Localization of DDAA-PICK1 in neurons.** (Related to Fig. 2. A) DDAA-PICK1 and WT-PICK1 co-cluster in neuronal dendrites. Cultured neurons were transfected with mCherry-WT-PICK1 (red) and GFP-DDAA-PICK1 (green). Images show a dendritic region representative of three independent experiments. (B) DDAA-PICK1 and WT-PICK1 show a similar degree of colocalization with PSDs. Cultured neurons were transfected with GFP-WT-PICK1 or GFP-DDAA-PICK1 (green) and stained with anti-Homer antibody to label PSDs (red). Graph shows Pearson's coefficients for GFP-PICK1–Homer colocalization (*n* = 3 independent experiments [18 cells in total]; two-tailed *t* test. (C) DDAA-PICK1 and WT-PICK1 show a similar degree of colocalization with early endosomes. Cultured neurons were transfected with GFP-WT-PICK1 or GFP-DDAA-PICK1 (green) and stained with anti-EEA1 antibody to label early endosomes (red). Graph shows Pearson's coefficients for GFP-PICK1–EEA1 colocalization; *n* = 3 independent experiments [18 cells in total]; two-tailed *t* test. (D) DDAA-PICK1 and WT-PICK1 show a similar degree of colocalization with recycling endosomes. Cultured neurons were transfected with GFP-WT-PICK1 or GFP-DDAA-PICK1 (green) and stained with anti-Rab11 antibody to label recycling endosomes (red). Graph shows Pearson's coefficients for GFP-PICK1–Rab11 colocalization; *n* = 3 independent experiments [18 cells in total]; two-tailed *t* test; values represent means ± SEMs). Bars, 5 µm.

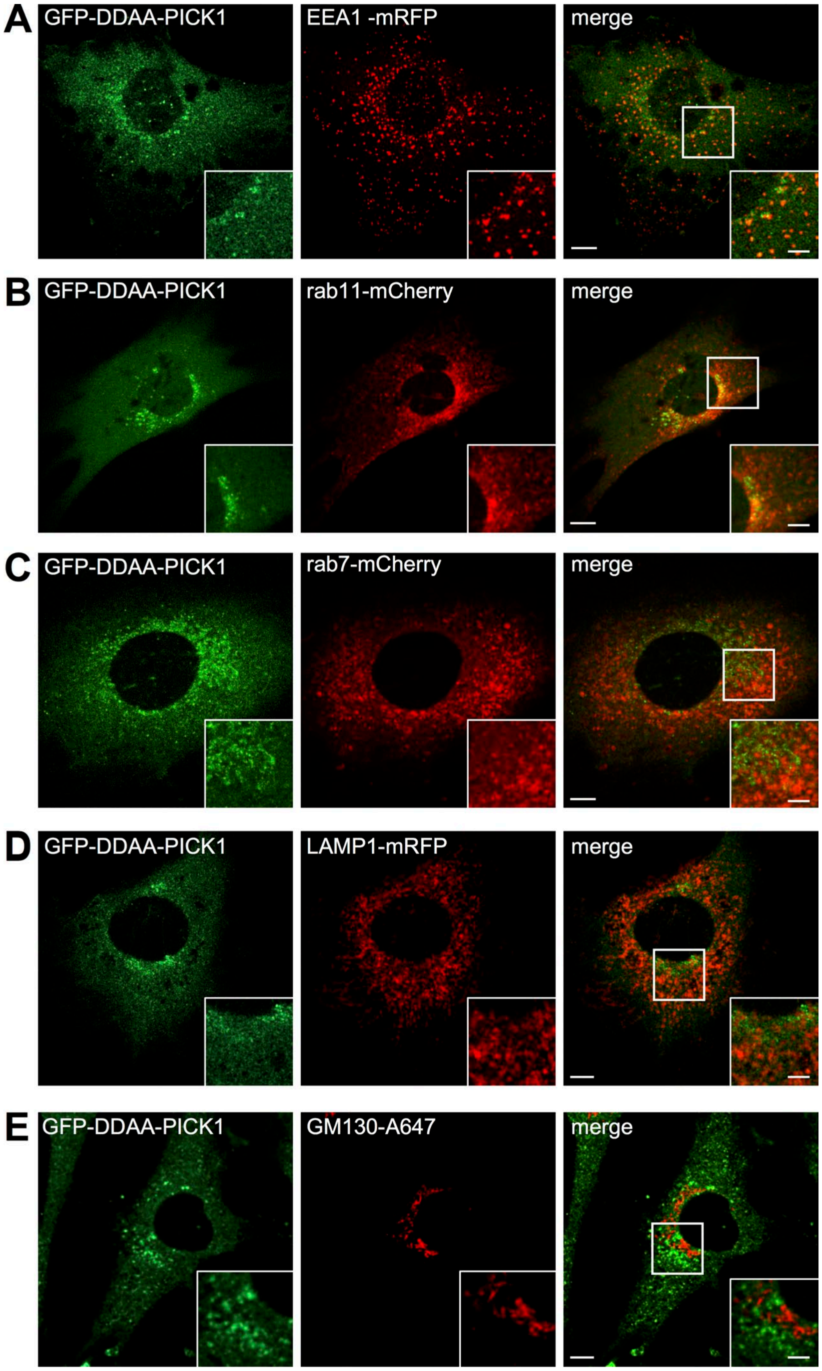

Figure S3. **Localization of PICK1 DDAA to endomembrane compartments.** Related to Fig. 3. (A–D) Mouse fibroblasts were cotransfected with GFP-DDAA-PICK1 and early endosome marker EEA1-mRFP (A); recycling endosome marker Rab11 (B); late endosome marker Rab7 (C); and lysosome marker LAMP1 (D) 40 h before confocal imaging. Note modest colocalization between individual PICK1 DDAA puncta and early endosomal marker EEA1, as well as recycling endosomal marker Rab11. DDAA-PICK1 puncta did not overlap with Rab7 and LAMP1. (E) Mouse fibroblasts were transfected with GFP-DDAA-PICK1 40 h before fixation with 4% PFA and immunostaining with anti-GM130 antibodies. Note no overlap between the DDAA-PICK1 signal and the Golgi marker. Bars: (whole cells) 10 µm; (magnified images) 5 µm.

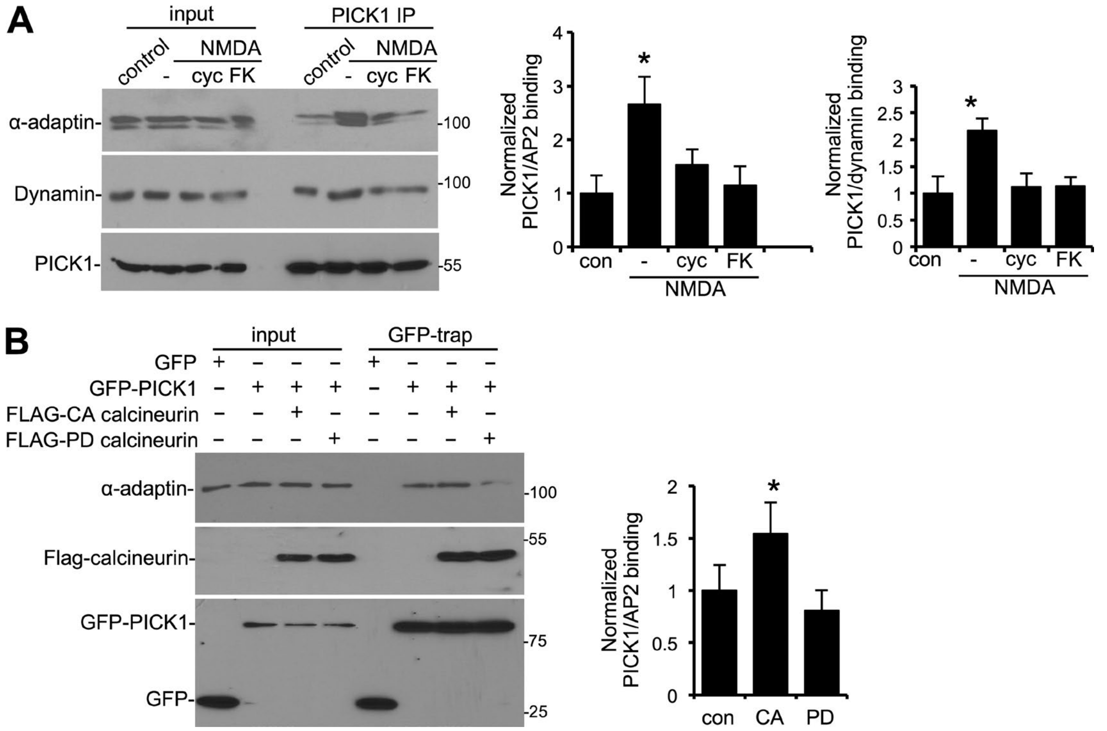

Figure S4. **Additional evidence of a role for calcineurin in regulating PICK1–AP2 and PICK1–dynamin interactions.** Related to Fig. 5. (A) FK506 has a similar effect as cyclosporin A in inhibiting the NMDA-stimulated increase in PICK1–AP2 and PICK1–dynamin interactions. Neurons were treated with 10 µM cyclosporin A (cyc), 1 µM FK506 (FK), or vehicle (-) 1 h before NMDA application. 4 min after return to normal medium, cell extracts were prepared for immunoprecipitation with anti-PICK1 antibodies. Proteins were detected by Western blotting. Graphs show quantification of PICK1–AP2 and PICK1–dynamin binding. (B) CA calcineurin causes an increase in PICK1–AP2 interactions. HEK293 cells were transfected with GFP, GFP-PICK1, FLAG CA calcineurin, or FLAG PD calcineurin as indicated. Cells were lysed and incubated with GFP-trap agarose. Graph shows quantification of GFP-PICK1 binding to endogenous AP2. (A and B) *n* = 5; *, $P < 0.05$; one-way ANOVA followed by Tukey's test; values are means ± SEMs.

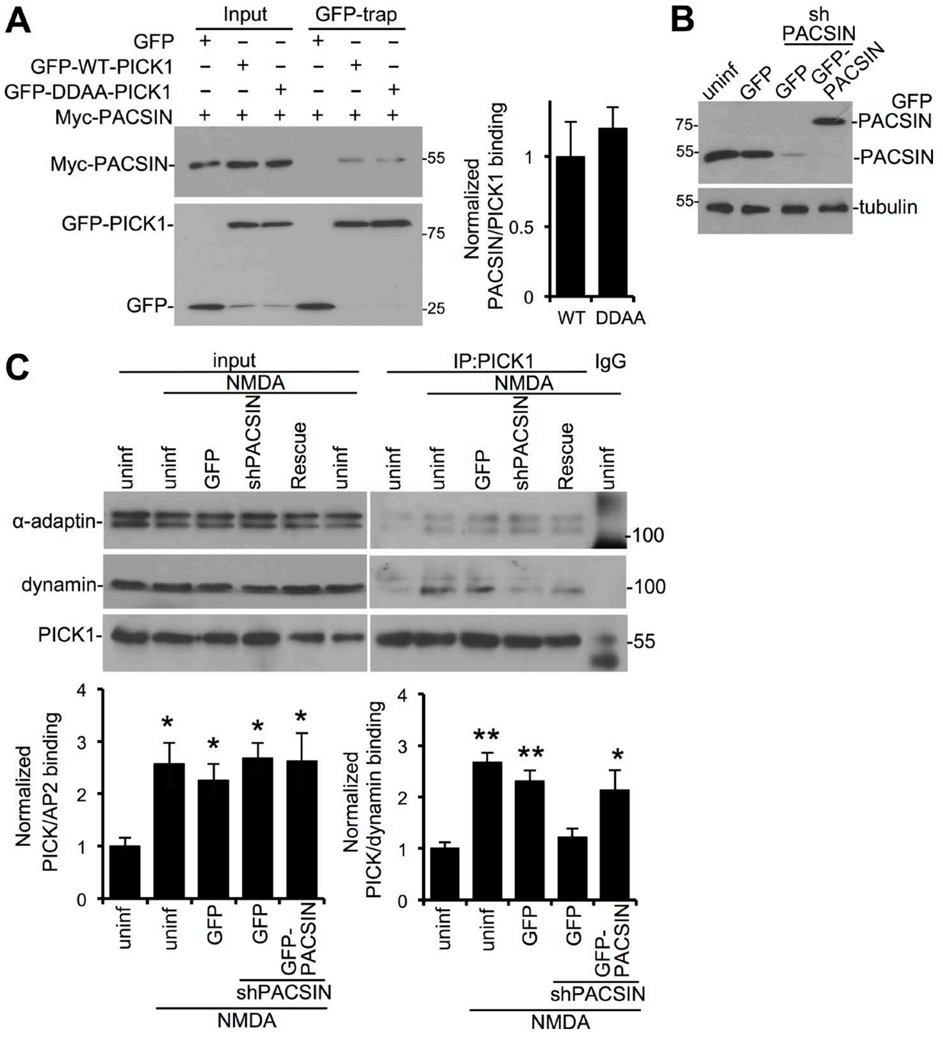

Figure S5. **PICK1–dynamin interaction, but not PICK1–AP2 interaction, involves PACSIN/syndapin.** Related to Fig. 5. (A) PACSIN/syndapin–PICK1 binding is unaffected by the DDAA mutation. HEK293 cells were transfected with GFP, GFP-WT-PICK1, GFP-DDAA-PICK1, or myc–PACSIN/syndapin as indicated. Cells were lysed and incubated with GFP-trap agarose. Graph shows quantification of GFP-PICK1 binding to myc–PACSIN/syndapin (*n* = 4; two-tailed *t* test; values represent means ± SEMs). (B) PACSIN/syndapin shRNA and coexpression of sh-resistant, GFP-tagged PACSIN/syndapin. Neurons were transduced with lentivirus expressing GFP; PACSIN/syndapin shRNA; and sh-resistant, GFP-tagged PACSIN/syndapin as indicated. (C) PACSIN/syndapin knockdown has no effect on the NMDA-stimulated increase in PICK1–AP2 interaction but blocks the increase in PICK1–dynamin interaction. Neurons transduced with lentiviruses as indicated were exposed to NMDA and then returned to normal medium for 4 min. Cell extracts were subjected to immunoprecipitation with anti-PICK1 antibodies. Graphs show quantification of PICK1–AP2 and PICK1–dynamin binding (*n* = 6; *, $P < 0.05$; **, $P < 0.01$).

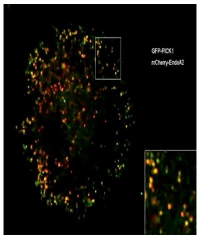

Video 1. **WT-PICK1 colocalized with endophilin-A2.** Related to Fig. 3 A. Dynamin TKO fibroblasts at 40 h after transfection with GFP-PICK1 and endophilin-A2–mCherry imaged on a spinning-disk confocal microscope. PICK1 colocalized with endophilin-A2 on the necks of arrested CCPs. Time-lapse images were captured every 4 s; video runs at 10 frames/s. Inset shows magnification of individual necks.

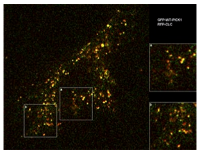

Video 2. **WT-PICK1 colocalized with clathrin.** Related to Fig. 3 B. Dynamin TKO fibroblasts at 40 h after transfection with GFP-PICK1 and mRFP–clathrin LC imaged on a spinning-disk confocal microscope. PICK1 colocalized with clathrin on arrested CCPs. Time-lapse images were captured every 4 s; video runs at 10 frames/s. Inset shows magnification of individual pits (a) in a juxtanuclear area and (b) in the cell periphery.

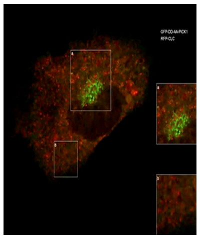

Video 3. **DDAA-PICK1 does not associate with clathrin but localizes to a juxtanuclear area.** Dynamin TKO fibroblasts at 40 h after transfection with GFP-DDAA-PICK1 and mRFP–clathrin LC imaged on a spinning-disk confocal microscope. DDAA-PICK1 does not colocalize with clathrin but is enriched in a juxtanuclear area. Time-lapse images were captured every 4 s; video runs at 10 frames/s. Inset shows magnification of (a) the juxtanuclear area and (b) the cell periphery.
